# Psychosocial and behavioural interventions for the negative symptoms of schizophrenia: a systematic review of efficacy meta-analyses

**DOI:** 10.1192/bjp.2023.21

**Published:** 2023-07

**Authors:** Matteo Cella, Safina Roberts, Matthias Pillny, Marcel Riehle, Brian O'Donoghue, John Lyne, Paul Tomlin, Lucia Valmaggia, Antonio Preti

**Affiliations:** Institute of Psychiatry, Psychology and Neuroscience, King's College London, UK; and South London and the Maudsley NHS Trust, UK; Clinical Psychology and Psychotherapy, Institute for Psychology, Universität Hamburg, Germany; Department of Psychiatry, University College Dublin, Ireland; and Centre for Youth Mental Health, University of Melbourne, Australia; Royal College of Surgeons in Ireland, Ireland; and Health Service Executive, Newcastle Hospital, Ireland; Institute of Psychiatry, Psychology and Neuroscience, King's College London, UK; Institute of Psychiatry, Psychology and Neuroscience, King's College London, UK; South London and the Maudsley NHS Trust, UK; and Katholieke Leuven Universitet, Belgium; Department of Neuroscience, University of Turin, Italy

**Keywords:** Psychosis, negative symptoms, psychosocial, schizophrenia, review

## Abstract

**Background:**

Currently there is no first-line treatment recommended for the negative symptoms of schizophrenia. Psychosocial and behavioural interventions are widely used to reduce the burden of negative symptoms. Meta-analytic studies have summarised the evidence for specific approaches but not compared evidence quality and benefit.

**Aim:**

To review and evaluate the evidence from meta-analytic studies of psychosocial and behavioural interventions for the negative symptoms of schizophrenia.

**Method:**

A systematic literature search was undertaken to identify all meta-analyses evaluating psychosocial and behavioural interventions reporting on negative symptom outcomes in people with schizophrenia. Data on intervention, study characteristics, acceptability and outcome were extracted. Risk of bias was evaluated. Results were summarised descriptively, and evidence ranked on methodological quality.

**Results:**

In total, 31 systematic reviews met the inclusion criteria evaluating the efficacy of negative symptom interventions on 33 141 participants. Exercise interventions showed effect sizes (reduction in negative symptoms) ranging from −0.59 to −0.24 and psychological interventions ranging from −0.65 to −0.04. Attrition ranged between 12% to 32%. Across the studies considered heterogeneity varied substantially (range 0–100). Most of the reviews were of very low to low methodological quality. Methodological quality ranking suggested that the effect size for cognitive remediation and exercise therapy may be more robust compared with other approaches.

**Conclusions:**

Most of the interventions considered had a small-to-moderate effect size, good acceptability levels but very few had negative symptoms as the primary intervention target. To improve the confidence of these effect sizes being replicated in clinical settings future studies should minimise risk of bias.

## Background

Negative symptoms are a cluster of psychosis symptoms characterised by a reduction or loss of normal functions. These include reductions in goal-directed and social behaviour, poor motivation, anhedonia, blunted affect and flattened speech.^1,2^ Negative symptoms negatively contribute to long-term outcomes in people with schizophrenia. They are highly prevalent in those with chronic illness,^3^ are associated with poor functional outcomes,^4^ reduced day-to-day activity and often lead to lower quality of life and reduced psychosocial functioning.^5,6^

Intervention development to date has strongly focused on positive symptoms and produced effective treatments.^7,8^ However, it is apparent that positive symptoms remission often does not correspond with a reduction of negative symptoms and/or improved recovery.^9,10^

In recent years it has been recognised that better treatments targeting negative symptoms are needed to improve long-term illness outcomes and recovery rates.^11^ To date attempts to develop and evaluate pharmacotherapy for negative symptoms have proven complex and showed, at best, modest benefits.^12,13^ Attempts at pharmacological augmentation of antipsychotic treatment have also shown little benefit compared with placebo, for example Deakin et al.^14^

## Psychosocial and behavioural interventions

Psychosocial and behavioural interventions for negative symptoms were developed and used alongside pharmacotherapies. In many cases, these interventions were not originally designed to target negative symptoms but adapted from other therapy targets (e.g. positive symptoms or depression). The landscape, however, is changing, with an increasing number of studies having negative symptoms as their primary intervention target. To date non-pharmacological approaches to treat negative symptoms have used methods consistent with different hypothesised treatment mechanisms and therapy techniques. Cognitive–behavioural therapy (CBT) approaches aim to challenge defeatist beliefs and generalised expectations of failure that might be associated with a lack of motivation and difficulties with pleasure experience.^15,16^ One of the initial studies in this area showed that the adapted model of CBT for negative symptoms was able to improve clients’ motivation and reduce apathy leading to improvements in functioning.^17^

Another approach increasingly used to target negative symptoms is cognitive remediation. Cognitive remediation may reduce negative symptoms by targeting the cognitive underpinning of negative symptoms including reward processing abnormalities, working memory, problem-solving and planning.^18–20^

More recent therapy developments for negative symptoms have seen the application of third-wave psychological treatments, exercise therapy and social skills training. Mindfulness-based interventions for negative symptoms include a behavioural component that is thought to encourage reactivity as well as increase anticipatory pleasure.^21^ Exercise-based interventions aim to improve motivation by using behavioural activation principles, which have shown promise in reducing negative symptoms.^22^ Finally, social skills training aims to support clients to develop expressive and receptive communication skills, enabling social contact and improving functioning in the community. These have been shown to reduce anhedonia, improve motivation and social engagement.^23^

## Aims

The increase in the number of studies reporting on negative symptom treatment outcomes has allowed, more recently, for the results to be aggregated in systematic reviews and evaluated with meta-analyses. However, the studies considered tend to have a high degree of heterogeneity for intervention and type of outcome, with negative symptoms only rarely evaluated as the primary outcome.^24^ Further, these reviews can vary in the information provided on intervention acceptability indicators (e.g. attrition rates). This is particularly important when considering treatment recommendations but also for efficacy trials, given that treatment retention in research studies can be problematic for people with schizophrenia.^25^

With these limitations in mind, it may be difficult to use the information in the literature to inform clinical guidelines for the treatment of negative symptoms. The aim of this review is to synthesise and appraise the evidence collated by existing meta-analyses on the efficacy of psychosocial and behavioural interventions for the negative symptoms of schizophrenia to guide clinical decision-making, guideline recommendations and future study approaches.

## Method

This review was conducted in line with the Preferred Reporting Items for Systematic Reviews and Meta-Analyses (PRISMA) guidelines for systematic reviews and meta-analyses.^26^ The protocol was registered on 14 August 2020 on PROSPERO (https://www.crd.york.ac.uk/prospero/display_record.php?RecordID=186496ID=CRD42020186496). The PICO framework was used to describe the elements of the review.^27^

### Participants

Participants were adults (18 years and over), with a diagnosis of schizophrenia or schizoaffective disorder. No restrictions were placed on the illness duration or severity.

### Interventions

Psychosocial and behavioural interventions were defined as any intervention that promoted physical and mental well-being. Interventions could be delivered either individually or in a group setting and could be offered in addition to treatment-as-usual, including pharmacotherapy. Only interventions offering more than one session were considered. Interventions using devices to alter brain functioning such as brain stimulation (e.g. repetitive transcranial magnetic stimulation or transcranial direct current stimulation) were not included.

### Control group

Any control groups including usual care, no intervention or other interventions including pharmacological interventions were included.

### Study design and outcomes

Studies were meta-analyses, including network meta-analyses, considering randomised controlled trials (RCTs) where the primary or secondary outcome was a validated measure of negative symptoms for people with schizophrenia. We only included meta-analyses where appropriate methods for statistical computation of results were used and reported (e.g. using standardised mean difference for measures of effect). To determine the acceptability of the interventions, we collected information on people who dropped out of the study. We also evaluated attrition bias and how data from participants who dropped out or failed to complete assessment measures was handled.

### Search strategy

A systematic search of the literature was performed in the following electronic databases: PsycINFO, EMBASE and Medline, using the OVID interface to find relevant studies, in addition to The Cochrane Library of Systematic Reviews. The search was restricted to systematic reviews including a meta-analysis; published in peer-reviewed journals; considering only RCTs. Publications from January 1980 to June 2022, limited to English language, were included in the search.

The search strategy was developed and adapted to fit the requirements for each of the databases. MESH and index terms of the following keywords were combined: (a) ‘psychosis’ ‘schizophrenia’, (b) ‘negative’, (c) ‘psychosocial’, and (d) ‘systematic review’. Relevant variations, synonyms and truncations were also included (see Supplementary Appendix 1 available at https://doi.org/10.1192/bjp.2023.21 for completed search strategy). EndNote was used to manage records throughout the review process.

### Study inclusion/exclusion criteria

Systematic reviews were included if they met the following inclusion criteria:

- samples were at least 75% participants with schizophrenia spectrum diagnosis;
- reported on a psychosocial and behavioural intervention;
- considered only RCTs;
- reported the effect of the intervention on a validated negative symptoms outcome;
- reported the comparison effect sizes between treatment and control condition;
- the effect size reported was calculated using meta-analytic procedures on at least two independent studies.

### Quality rating

All the included reviews were independently rated for methodological quality by two reviewers using the AMSTAR tool. This is a validated tool to assess the methodological quality of systematic reviews.^28^ The tool includes 16 items; individual items are combined to give an overall rating related to study quality at one of four levels: (a) critically low quality, (b) low quality, (c) moderate quality, (d) high quality.

### Data extraction

After duplicate citations were removed, a two-part screening and extraction process was conducted. First, titles and abstracts were independently screened by S.R., MR, M.P. and P.T. Where there were discrepancies, these were discussed and resolved by M.C.

Data extraction was performed by two authors pairs: S.R. and P.T. (pair 1) and M.R. and M.P. (pair 2). At least two authors extracted data independently from papers and performed data checks. Any disagreement was resolved in discussion with a third author (M.C.). Information was extracted using a data extraction template based on the PICO (patient or population; intervention; comparison intervention or condition; outcome) framework.^29^

The information extracted included: inclusion criteria, number of studies, sample diagnoses, total number of participants considered, age range and/or mean, type of intervention, length of interventions (on average), control group, people who dropped out of the study, measure of negative symptoms used, other outcome measures included in the review, statistical analyses performed, mean effect size and heterogeneity. All information was extracted from the review papers considered. To aid comparability, effect sizes are represented in a way that a negative number shows advantage of the active over the control condition (i.e. reduction of negative symptoms).

### Evidence ranking

Study risk of bias parameters were used to rank the available evidence based on their methodological quality (first criterion), number of participants considered (second criterion) and variability of intervention effect or statistical heterogeneity (third criterion). The results were presented graphically.

## Results

### Search outcome

**Fig. 1:**
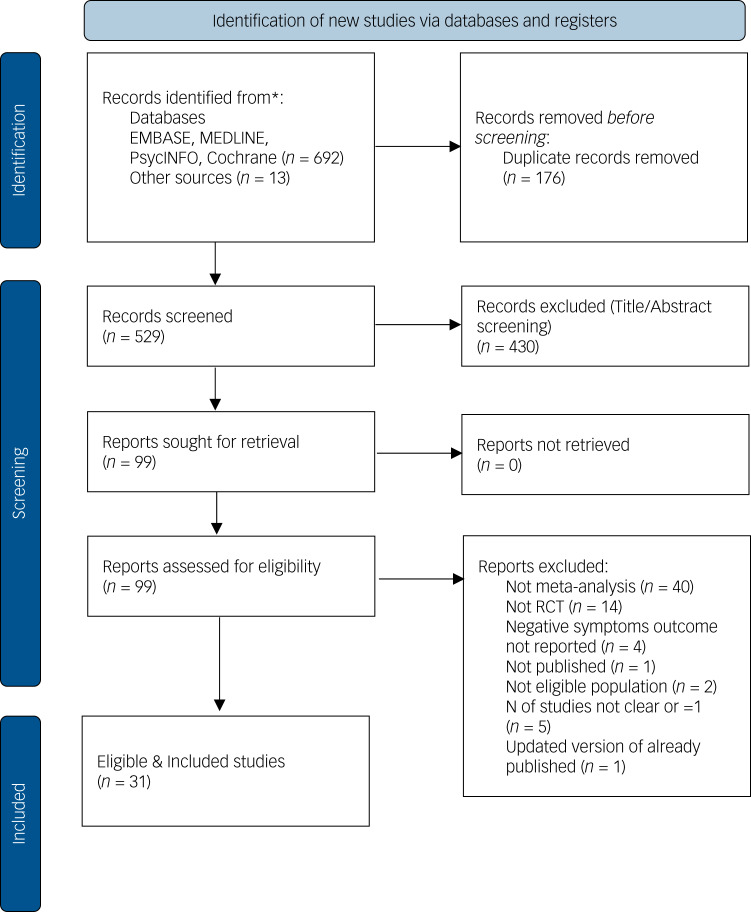
PRISMA flow diagram for identification, screening and eligibility of studies. RCT, randomised controlled trial.

The paper selection process is shown in in Fig. 1 (i.e. PRISMA flow chart). The initial search yielded 692 citations from which 31 meta-analyses reports met inclusion criteria and were included.

### Descriptive analysis of systematic reviews

#### Studies general characteristics

Supplementary Table 1 provides a summary of the 31 reviews included. These studies includes 33141 participants. Of these, 23 evaluated psychological therapies, 5 exercise therapy, 2 music therapy and 1 multiple approaches. The number of studies included in each review ranged between 2 and 95 with an average of 15.1 studies (median, 10). The objectives of the reviews varied with eight focusing specifically on negative symptoms^16,30–36^ and the rest investigating negative symptoms alongside other mental and physical health outcomes. Twenty-two reviews evaluated a single intervention approach while nine compared multiple therapeutic approaches.^31,37–45^

#### Population

Participants number, used for the estimation of negative symptom treatment effect, ranged from 67 to 2878 and the average participants number was 872.1. Age ranged from 18 to 78 years. Most of the studies did not distinguish participants based on their illness stage (e.g. early or chronic phase). One study included only individuals with treatment-resistant schizophrenia.^42^

#### Interventions

Intervention approaches are described in Supplementary Table 1. Six reviews considered forms of exercise therapy including yoga, meditation and tai-chi,^30,32^ anaerobic and aerobic exercise,^33^ mind–body exercise and resistance training.^31,34^ Studies generally defined exercise as any activity aimed at improving or maintaining physical fitness.^46^

Two studies evaluated the effect of music therapy,^47,48^ considered as an intervention aiming to promote health in the context of music experience.^47^ The studies considered included active (e.g. music making) and receptive music (e.g. music listening techniques) therapy methods.

Psychological therapies included CBT interventions,^16,49^ social skills training,^50,51^ acceptance and mindfulness-based approaches^38,39^ and family interventions.^52^

Three examined specifically cognitive remediation,^36,43,53^ two studies reported on group therapies,^37,41^ two reported on family-based therapy,^31,54^ and one study focused on integrated neurocognitive therapy.^55^

Three studies compared multiple psychological approaches^31,35,43^ with Turner et al^43^ reporting on multiple therapies including befriending, CBT, cognitive remediation, psychoeducation, social skills training and supportive counselling.

#### Control group characteristics

Most exercise therapy studies included treatment-as-usual (TAU) or waitlist as a control condition (see Supplementary Table 1); three studies also considered active control conditions, for example Cramer et al,^30^ Vogel et al^34^ and Riehle et al.^35^ For studies evaluating CBT and mindfulness-based interventions, control conditions ranged from active controls, non-active controls, waitlist, TAU or standard care. Two studies conducted different meta-analytic comparisons of psychological approaches, with each intervention being compared with the other interventions pooled.^43,45^ The reviews evaluating group therapies considered active control groups (psychotherapeutic treatments), passive control groups (waitlist controls, attention control) and TAU.^37,41^ For social skills training the majority of the control conditions were active controls and TAU.^50,51^ For cognitive remediation the control groups were TAU alone and TAU with the addition of an active control.^36^ All the studies evaluating family intervention had TAU as their control group.

#### Outcome measures

The most frequent negative symptoms outcome measure was the Positive and Negative Syndromes Scale (PANSS, 96.7%),^56^ followed by the Scale for Assessment of Negative Symptoms (SANS; 43.4%)^57^ and the Brief Psychiatric Rating Scale (BPRS, 36.6%).^58^ Other measures used included the Brief Symptom Inventory,^59^ Negative Symptoms Assessment,^60^ Brief Negative Symptom Scale^61^ and Comprehensive Psychopathological Rating Scale.^62^

### Effect of interventions on negative symptoms

#### Exercise interventions

Results of five of the meta-analyses suggested that exercise interventions had a significant and positive effect in reducing negative symptoms, with a small-to-medium effect size. One review did not report a significant effect of exercise on negative symptoms^30^ (see Table 1).

**Table 1:** Meta-analyses results on the effect of exercise interventions on negative symptoms

Study	Control group	Number of participants	Model	Effect size	95% CI		*P*	*I*^2^, %
					Lower	Upper		
Sabe et al 2020	AC	954	RE	SMD = −0.24	−0.43	−0.06	0.01	45
Vogel et al 2019	AC	1249	RE	*g* = −0.43	−0.20	−0.67	0.00	76
Sabe et al 2019	AC	1081	RE	SMD = −0.36	−0.58	−0.15	0.00	62
Lutgens et al 2017	TAU	−	RE	*g* = −0.36	−0.71	−0.01	0.04	55
Firth et al 2015	AC	659	RE	SMD = −0.44	−0.78	−0.09	0.01	0
Cramer et al 2013	TAU	198	RE	SMD = −0.59	−1.87	0.69	0.36	80

*g*, Hedges *g*; AC, any comparator; RE, random effects; SMD, standardised mean difference; TAU, treatment-as-usual.

Two reviews evaluated the effect of the intervention compared with TAU and active controls separately. Cramer et al^30^ did not find a significant effect for yoga when compared with either TAU or active controls.^30^ Lutgens et al^31^ reported that the beneficial effect of exercise on negative symptoms was observed when the comparison group was TAU but there was no significant difference between exercise and active controls. Vogel et al^34^ also reported that exercise interventions were only effective when compared with TAU.

Three of the reviews considered mind–body exercises. One review evaluated yoga.^30^ Two reviews included several mind–body exercise approaches including tai-chi, yoga and mindfulness.^32,34^ Subgroup analyses revealed a small but significant effect favouring yoga, with high heterogeneity, which disappeared when studies at high risk of bias were excluded.^32^

Sabe et al^33^ found that the effect of physical exercise on negative symptoms was driven by aerobic exercise (standardised mean difference (SMD) = −0.31, 95% CI −0.54 to −0.09; *P* < 0.01) but a different review showed this result may be affected by high risk of bias.^34^

Cramer et al^30^ showed no short-term benefits (i.e. 12 weeks after randomisation) on negative symptoms when comparing yoga to exercise or TAU.^30^ Sabe et al^32,33^ reported intervention lengths between 14.5 and 20.8 weeks, however, Vogel et al^34^ showed that intervention length does not influence efficacy.

#### Music therapy

The two studies that examined music therapy showed moderate effect sizes in reducing negative symptoms compared with TAU (see Table 2). In a study by Geretsegger et al,^47^ music therapy combined with standard care showed a significant effect after intervention on negative symptoms when compared with TAU (SMD = −0.55, 95% CI −0.87 to −0.24, *P* < 0.001). Jia et al^48^ found that music therapy was able to reduce negative symptoms compared with TAU (SMD = −0.61, 95% CI −0.80 to −0.42, *P* < 0.05) and that interventions lasting >3 months were more effective in negative symptom reduction compared with those lasting < 3 months. Lutgens et al^31^ also evaluated arts-based treatments (including music therapy), which had no effect on negative symptoms, but in a sensitivity analysis they found a moderate effect of music therapy on reducing negative symptoms compared with TAU.

**Table 2:** Meta-analyses results on the effect of psychological interventions on negative symptoms

Study	Control group	Participants, *n*	Model	Effect size	95% CI		*P*	*I*^2^, %
					Lower	Upper		
Cognitive–behavioural therapy								
Jones, 2018^44^	TAU	729	FE	MD = −3.35, SMD = −0.49^a^	−3.84	−2.85	<0.001	72.19
	TAU	1087	FE	MD = −1.43, SMD = −0.17^a^	−1.94	−0.93	<0.001	84.37
	TAU	1436	FE	MD = −1.47, SMD = −0.16^a^	−1.94	−0.99	<0.001	89.01
	TAU	231	RE	MD = −4.11, SMD = −0.08^a^	−10.4	2.17	0.20	95.08
Jones, 2018^45^	OPT	581	FE	*d* = −0.06	−0.32	0.20	>0.05	11.24
	OPT	45	RE	*d* = −0.08	−0.67	0.50	>0.05	0.00
	OPT	68	FE	*d* = −0.12	−0.36	0.59	>0.05	100
Jauhar, 2014^49^	AC	−	RE	*g* = −0.13	−0.25	−0.01	0.03	47.70
Lutgens, 2017^31^	CBT vs AC	−	RE	SMD = −0.34	−0.55	−0.12	0.00	73.60
Polese, 2019^42^	TAU	800	FE	SMD = 0.08^c^	−0.06	0.21	0.286	0.00
Riehle, 2020^35^	TAU	278	RE	*g* = −0.24	−0.47	0.004	−	0.00
	CR	238	RE	*g* = 0.12	−0.14	0.37	0.36	0.00
Sarin, 2011^64^	OPT	461	RE	*g* = −0.10	−0.28	0.09	0.31	24.00
	TAU	318	RE	*g* = −0.21	−0.43	0.01	0.07	0.00
Turner, 2014^43^	OPT	−	RE	*g* = 0.04^c^	−0.09	0.16	>0.05	0.00
Velthorst, 2015^17^	TAU	2312	RE	*g* = 0.09^c^	−0.03	0.214	0.13	62.70
	TAU		RE	*g* = 0.16^c^	−0.10	0.41	0.23	−
Wykes, 2008^63^	TAU	1268	RE	*d* = 0.44^c^	0.17	0.70	<0.05	118.1
Music therapy								
Jia, 2020^48^	TAU	775	RE	SMD = −0.61	−0.80	−0.42	<0.001	38.0
Geretsegger, 2017^47^	TAU	177	FE	SMD = −0.55	−0.87	−0.24	<0.001	−
Acceptance and mindfulness-based								
Cramer, 2016^38^	TAU	111	RE	*g* = −0.27	−0.72	0.17	0.23	14.00
Hodann-Caudevilla, 2020^65^	AC	506	RE	*g* = 0.40^c^	0.29	0.51	<0.01	82.50
Jansen, 2020^39^	AC	1268	RE	*d* = −0.24	−0.44	−0.03	0.02	23.00
Liu, 2021^67^	TAU	460	RE	*g* = −0.53	−0.72	−0.35	<0.001	0.00
	TAU	460	RE	*g* = −0.59	−0.78	−0.41	<0.001	0.00
Tonarelli, 2016^66^	TAU	67	FE	SMD = 0.65^c^	0.17	1.13	0.008	0.00
Group								
Burlingame, 2020^37^	AC	4156 (total)	RE	*g* = 0.27^c^	0.15	0.40	<0.00	57.00
Orfanos, 2015^41^	TAU	893	RE	*d* = −0.37	−0.60	−0.14	<0.00	59.80
	AC	783	RE	*d* = −0.09	−0.36	0.19	0.54	68.50
Social skills								
Kurtz, 2008^50^	AC	363	FE	*d* = 0.40^c^	0.19	0.61	<0.05	−
Lutgens, 2017^31^	AC	−	RE	SMD = −0.31	−0.45	−0.17	<0.05	−
Turner, 2014^43^	AC	3295	RE	*g* = 0.27^c^	0.01	0.53	<0.05	53.80
Turner, 2018^51^	AC	−	RE	*g* = 0.19^c^	0.04	0.34	<0.05	18.65
Cognitive remediation								
Turner, 2014^43^	OTP	−	RE	*g* = −0.14^b^	−0.39	0.06	>0.05	40.99
Cella, 2017^36^	AC	2511	FE	*g* = −0.34	−0.42	−0.27	<0.01	−
	AC	2511	RE	*g* = −0.35	−0.44	−0.25	<0.01	28
Lejeune 2021^53^	AC	2581	RE	*g* = 0.16^c^	0.04	0.29	0.012	42.88
Family-based								
Ma, 2020^54^	TAU	158	RE	MD = −4.35, SMD = −0.53^a^	−5.62	−3.08	<0.05	31
Rodolico, 2022^52^	TAU	2313	RE	SMD = −0.36^b^	−0.51	−0.21	<0.01	65
Lutgens, 2017^31^	AC	−	RE	SMD = −0.19	−0.70	0.34	>0.05	−
Integrated								
De Mare, 2018^55^	TAU	217	FE	MD = −2.47	−4.11	−0.83	<0.01	0

AC, any comparator; *d* = Cohen's d; FE, fixed effects; *g* = Hedges’ *g*; MD, mean difference; OPT, other psychological therapies; RE, random effects; SMD, standardised mean difference; TAU, treatment-as-usual.

a. SMD calculated see Supplementary Appendix 4 for details.

b. Review presented negative value as favouring control condition.

c. Review presented positive value as favouring experimental condition.

#### CBT

The effect of CBT was examined in ten studies (see Table 2). Overall, these studies showed small-to-moderate effects of CBT in reducing negative symptoms when compared with TAU and no differences between CBT and other psychological treatments.

Lutgens et al^31^ and Wykes et al^63^ showed CBT to be more effective compared with TAU with small effect sizes. The results by Velthorst et al^16^ and Wykes et al^63^ suggest that these effects are smaller and non-significant in studies with lower risk of bias. Jones et al^45^ found significant negative symptom reductions for CBT compared with TAU in studies using the PANSS as the outcome measure based on short- (up to 24 weeks), medium- (24–52 weeks) and long-term (over 52 weeks) follow-up periods, and no effect for studies using the SANS (only short-term follow-up available). Sarin et al^64^ found a small, approaching significance, effect at follow-up (3–15 months after treatment) but no immediate post-treatment effect.

In RCTs focusing on treatment-resistant schizophrenia, Polese et al^42^ did not find an effect of CBT compared with TAU. In RCTs including only patients with elevated negative symptoms before therapy, Riehle et al^35^ found a small, approaching significance, effect of negative symptom reduction for CBT compared with TAU. Secondary outcome analyses showed a small-to-moderate significant effect on motivational negative symptoms and no effect on expressive negative symptoms.

In meta-analyses comparing CBT with active controls or other psychological therapies, no study found a significant difference for the reduction of negative symptoms immediately post-treatment.^31,35,43,45,49^ Sarin et al^64^ found a small effect favouring CBT over other psychological treatments at follow-up.

#### Social skills training

All the four reviews evaluating social skills training^31,43,50,51^ found it to be effective in reducing negative symptoms. Turner et al^43^ also conducted analyses excluding studies with high risk of bias and showed this finding to be robust (*g* = −0.32, *P* < 0.05).

#### Acceptance and mindfulness-based

Five reviews reported on acceptance and mindfulness-based therapies. Four of these found significant small-to-moderate effects for reducing negative symptoms when comparing mindfulness-based therapies with TAU with^39,65^ and without an active control condition.^66,67^ No difference with TAU was found in a smaller meta-analysis by Cramer et al.^30^

#### Group therapies

Group therapy was investigated in three reviews.^31,37,41^ The results showed a small and significant overall effect of group therapies compared with controls post-treatment. Burlingame et al,^37^ conducted follow-up analyses showing that group social skills training and cognitive remediation had a small-to-medium significant reduction on negative symptoms (*g* = 0.23, 95% CI 0.03–0.34, *P* = 0.03; *g* = 0.56, 95% CI 0.29–0.84, *P* < 0.001), respectively.

#### Cognitive remediation

Three reviews investigated the effect of cognitive remediation.^36,43,53^ Cella et al^36^ and Lejeune et al^53^ showed significant small-to-moderate effects for cognitive remediation compared with controls at post-treatment (see Supplementary Table 1). Sensitivity analysis in Cella et al^36^ found that studies with higher methodological quality had a larger effect size compared with those with lower quality. Turner et al,^43^ found no significant difference between cognitive remediation and all other therapies on negative symptoms, and this remained non-significant after sensitivity analyses. Additionally, Riehle et al^34^ found no difference between cognitive remediation and CBT in reducing negative symptoms in RCTs in which patients had elevated negative symptoms before therapy.

#### Family-based therapy

The review by Ma et al^54^ found a significant effect of family-based therapies on negative symptoms whereas Lutgens et al^31^ found no effect regardless of the control comparison (see Supplementary Table 1). Long-term effects were not reported in either of the reviews. Rodolico et al^52^ found a significant overall effect on negative symptoms of family interventions compared with TAU. Their subgroup analyses with *k* ≥ 2 indicated superiority of ‘community-based supportive care interventions’, and the combination of family psychoeducation with patient behavioural skills training. Combinations with family behavioural skills training, mutual supportive skill training or emotional climate-focused interventions were not superior to TAU.

#### Other interventions

The review by De Mare et al,^55^ reported short-term effects of integrated neurocognitive therapy at 15 weeks post-treatment and showed a significant reduction of negative symptoms (see Supplementary Table 1) with this result maintained at 9–12-month follow-up.

Turner et al,^43^ found no evidence in support of befriending, psychoeducation or supportive counselling being superior to the other interventions pooled. Lutgens et al^31^ found a small beneficial effect for miscellaneous interventions on negative symptoms in comparison with TAU but not for active control.

### Acceptability

Nine out of the 31 reviews considered reported attrition rates (see Supplementary Appendix 2). For studies on exercise Firth et al described an attrition rate of 32%.^46^ Vogel et al^34^ reported the average attrition rate for group interventions was 15.2%, which was marginally higher than the average drop-out rate of participants in the TAU group (14.64%). Burlingame et al^37^ reported an average attrition rate of 12%, with no difference between the intervention and the control groups for group psychotherapy. Riehle et al^35^ reported an average attrition rate of 16% in CBT treatment arms, 20% in cognitive remediation, and 11% in TAU. Jones et al^45^ reported a drop-out rate of 14% for CBT plus standard care and for 13% for standard care. Sarin et al^64^ reported that the overall attrition rate was 14% at post-treatment and 17% at follow-up. Cella et al^36^ reported no differences in attrition rates between the intervention and the control group. Hodann-Caudevilla et al^65^ reported an average 14% attrition rate in mindfulness-based intervention treatment arms. Finally, De Mare et al^55^ reported attrition rates for integrated therapies to be below 15% at the end of treatment as well as at follow-up.

### Attrition bias

Most reviews considered attrition bias risk as part of their quality assessment. In Sabe et al^32,33^ studies with high attrition risk were considered and sensitivity analyses performed. Similarly, Turner et al^43,51^ analysed studies with a high and low risk of bias separately. Orfanos et al^41^ also excluded those studies that were rated high on risk of bias for drop-out. The review by Cramer et al^38^ reported that two out of the three papers considered used an intention-to-treat analysis. Likewise, Riehle et al^35^ reported that one of the CBT RCTs did not conduct an intent-to-treat analysis. Jansen et al^39^ also reported that one of the studies considered had a high risk of attrition bias although it did not affect the results. Jones et al^45^ reported five of the studies considered were at high risk of bias.

A few reviews excluded studies that did not meet pre-specified requirements around risk of bias. Jauhar et al excluded studies with attrition above 20%.^49^ Jones et al^40^ excluded studies with attrition above 40%. Ma et al excluded studies with one or more of the Cochrane Risk of Bias items rated as high, which eliminated those studies with a high risk of attrition bias.^54^ Similarly, the review by Geretsegger et al^47^ only included studies with an overall low risk of bias.

### Quality ratings

The AMSTAR ratings ranged from critically low quality to moderate quality (see Supplementary Appendix 3) with most of the reviews rated as critically low (71%). Only one review was of moderate quality.^47^

### Evidence ranking

**Fig. 2:**
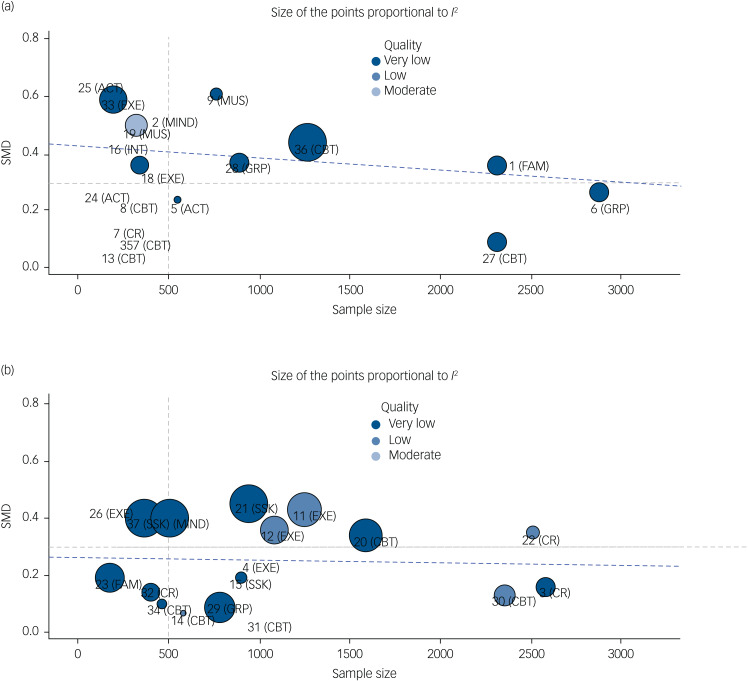
(a) Shows studies ranking compared with treatment-as-usual according to: (i) methodological quality (study dot colour); (ii) number of participants considered (*x*-axis); (iii) heterogeneity (size of the dot). Study effect size is represented on the *y*-axis with standardised mean difference (SMD). (b) Shows studies ranking compared with active treatment according to: (i) methodological quality (study dot colour); (ii) number of participants considered (*x*-axis); (iii) heterogeneity (size of the dot). Study effect size is represented on the *y*-axis with SMD. Note: 1^52^; 2^67^; 3^53^; 4^33^; 5^39^; 6^37^; 7/8^35^; 9^48^; 10^65^; 11^34^; 12^32^; 13^42^; 14^45^; 15^51^; 16^55^; 17^44^; 18/20/21/23^31^; 19^47^; 22^36^; 24^38^; 25^66^; 26^46^; 27^17^; 28/29^41^; 30^49^; 31/32^43^;33^30^; 34/35^64^; 36^62^; 37^50^. ACT, acceptance and commitment therapy; CBT, cognitive–behavioural therapy; CR, cognitive remediation; EXE, exercise therapy; FAM, family therapy; GRP, group therapy; INT, integrated therapy; MIND, mindfulness; SSK, social skills training.

Figure 2 shows the ranking of the included studies according to:

- methodological quality (study dot colour);
- number of participants considered (X axis);
- heterogeneity (size of the dot).

Effect sizes are represented on the y-axis. Medium effect size threshold (i.e. 0.3) and sample size (i.e. *n* = 500) are overlayed to aid the interpretation. For the size of the evidence considered, methodological quality and heterogeneity, the studies by Cella et al^36^ on cognitive remediation and Vogel et al^34^ and Sabe et al^32^ on exercise-based therapies appear to present the most robust results.

## Discussion

### Main findings

This study is the first, to the authors’ knowledge, to synthesise the evidence collated by existing meta-analyses on the efficacy of psychosocial and behavioural interventions for the negative symptoms of schizophrenia. It considered 31 peer-reviewed meta-analyses including > 30 000 participants.

Overall, the studies included indicate that psychosocial and behavioural interventions can reduce negative symptoms. This is despite heterogeneity across reviews in terms of the treatment conditions, duration and intensity but also outcome assessment and study design.

### Interpretation of our findings on interventions

Psychological therapies were the largest category, with the most evidence available for cognitive-based approaches, however, only one review considered treatments with negative symptoms as a primary intervention target. This is a reflection of the evidence base for psychological interventions in psychosis, which, until recently, has focused predominantly on positive symptoms.^17^ For CBT interventions, most reviews reporting a positive outcome found small effect sizes, and outcomes varied depending on the control condition, and the time point at which outcomes were measured. The risk of bias was also high for most of these studies. Currently, CBT is recommended in some clinical guidelines (e.g. the UK National Institute for Health and Care Excellence guidelines) for the treatment of positive symptoms. Although our study shows that this approach may lead to small improvements in negative symptoms, previous research suggested that the effect of CBT beyond positive symptoms may be limited.^68^ Further research with methodologically rigorous trials with targeted CBT interventions, predefined assessment, clear treatment dosage and long-term follow-up would be needed to expand the evidence base.

Four out of six reviews on exercise intervention reported these interventions can reduce negative symptoms. These reviews also had low methodological quality and large heterogeneity. Interestingly, effect sizes varied according to the type of exercise included, with favourable outcomes for mind–body exercise and aerobic exercise. Exercise-based therapies may reduce negative symptoms by promoting behavioural activation and offering opportunities for enjoyable activities. If these were found to be important therapy elements, it may be possible to incorporate these more widely for the management of negative symptoms. Other elements such as the social aspect of exercise may be also contributing to negative symptoms reduction.^22^ Importantly, there may be additional benefits of exercise therapy contributing to physical well-being. Research has shown that people with schizophrenia are more likely to have physical health comorbidities, which are often associated with lifestyle behaviours including weight gain, poor diet, and smoking, in addition to the side-effects from medication.^69–71^

Results from acceptance and mindfulness-based approaches were mixed, with reviews reporting small-to-moderate significant effects. This was similar to the observed effects of social skills training and group interventions. Importantly most of the reviews in these categories were rated as of critically low methodological quality and included a very small number of studies and participants, for example Tonarelli et al.^66^

The results for cognitive remediation, family-based and integrated therapies are promising and showing that each of these approaches have moderate and significant effects on negative symptoms. Although the evidence base for family-based and integrated therapy it is still in its infancy, the evidence for cognitive remediation appears more developed and robust although only a few studies considered negative symptoms as their primary intervention target. Overall, the effect sizes for the psychological therapies considered are similar. This is consistent with literature suggesting that the effects of psychological treatments are largely driven by common factors (e.g. empathy, alliance, collaboration) and that the comparisons of different forms of psychotherapy often result in non-significant differences, and contextual and relational aspects often mediate or moderate outcomes.^72^

Research has shown that engaging people with schizophrenia in psychological therapy can be challenging, with an average of 16% of people discontinuing CBT.^73^ The clinical presentation of people with negative symptoms including reduced motivation and apathy may make therapy attendance even more difficult^1,74^ and therefore interventions with good acceptability is important.

### Attrition and negative symptom severity

Only nine of the 31 included reviews commented on attrition. The remaining reviews either did not report on attrition or noted that the included studies had insufficient information on drop-out rates. This is an issue future reviews and clinical trials should consider carefully as attrition could have an impact on intervention implementation.^75^ The limited evidence collated in this study suggests that exercise, group therapy, CBT and cognitive remediation have similar attrition rates to those reported in control groups (between 10% to 32%). A further aspect which may compound attrition and received limited attention in the studies considered is negative symptom severity. Evidence of intervention efficacy in individuals with primary and secondary negative symptoms would be of clinical relevance as presentations and settings (e.g. in-patient or out-patient) in which interventions may be delivered are likely to be different.^1^ Interventions may also need to be adapted to address more rooted motivational and pleasure experience difficulties in those with primary negative symptoms who may require therapies to be more engaging and feel more relevant for their goals.

Most of the studies considered in the reviews did not have negative symptoms as their primary outcome. This is important when interpreting results, as many interventions were not specifically designed to treat negative symptoms and analyses may have not been sufficiently powered to assess efficacy for this outcome.

### Assessing negative symptoms

More than 95% of the reviews considered used the PANSS as their method of assessment for negative symptoms. Although popular, due to its capacity of assessing multiple symptoms domains, one of the PANSS key limitations is considering negative symptoms as a unitary construct. Increasing evidence suggests that negative symptoms are multidimensional and expressive (e.g. diminished expression) and experiential (e.g. motivational difficulties and anhedonia) symptoms should be considered separately.^76,77^ More recent empirical accounts advocate for considering negative symptoms as five distinct dimensions including blunted affect, alogia, anhedonia, avolition and asociality.^78^ These dimensional accounts are reflected in a new generation of assessment tools that are increasingly used in research studies such as the Clinical Assessment Interview for Negative Symptoms^79^ and the Brief Negative Symptom Scale.^80^ There is increasing recommendation for these measures to be used in intervention trials targeting negative symptoms as they could offer an insight into the clinical dimensions targeted by the intervention.^81^

The methodological rigour assessed by the AMSTAR tool was largely low to critically low. This tool is specifically designed to assess meta-analyses and as such reflects the quality of the synthesis made, not of the individual studies.

### Strengths and limitations

This review was conducted in line with best practice for the systematic evaluation of reviews and followed the PICO framework.^82,83^ We also used recommended practices for minimising the risk of biases, for example by including independent raters to screen studies, extract information and rate the methodological quality of reviews.

Limitations include the heterogeneity of samples, protocols and outcome measures considered across all the reviews that reduced the extent of the comparisons possible. For papers rating, for selection and risk of bias, we did not evaluate rater agreement and instead used a third rater to resolve any disagreement. We did not perform an evaluation of treatment effects at follow-up as the follow-up periods (and reporting) between studies had high variability.

### Implications

Overall, this review highlights limited robust evidence for psychosocial and behavioural interventions for treatment of negative symptoms. This is a significant gap that has an impact on the long-term quality of life and functioning outcomes for people with schizophrenia but also clinical service provision and resources.^5^ Efforts should be directed to further develop and evaluate interventions whose primary target are negative symptoms, including interventions targeting distinct components such as motivation or pleasure experience difficulties based on individual case formulations.^84^ Intervention development may also take advantage of digital technology tools that may facilitate or complement the delivery of interventions; with a recent example of this being a virtual-reality-supported psychological-targeted intervention for negative symptoms.^85^

Optimally intervention trials should be well defined in terms of their model of intervention, mechanics dosage, comparison conditions and provide information on the long-term benefits and cost–benefit. The results of the current study map the state of the evidence and indicate some interventions approach with the potential to be further developed, evaluated and used routinely in clinical practice.

## Data Availability

The data supporting the findings of this study are largely available in the online appendices. Additional data requests can be forwarded to the corresponding author.

## Supplementary material

For supplementary material accompanying this paper visit https://doi.org/10.1192/bjp.2023.21. click here to view supplementary material

## References

[1] Galderisi S, Mucci A, Buchanan RW, Arango C. Negative symptoms of schizophrenia: new developments and unanswered research questions. Lancet Psychiatry 2018; 5: 664–77. 29602739 10.1016/S2215-0366(18)30050-6

[2] Marder SR, Galderisi S. The current conceptualization of negative symptoms in schizophrenia. World Psychiatry 2017; 16: 14–24. 28127915 10.1002/wps.20385 PMC5269507

[3] Bobes J, Arango C, Garcia-Garcia M, Rejas J. Prevalence of negative symptoms in outpatients with schizophrenia spectrum disorders treated with antipsychotics in routine clinical practice: findings from the CLAMORS study. J Clin Psychiatry 2010; 71: 280–6. 19895779 10.4088/JCP.08m04250yel

[4] Rabinowitz J, Levine SZ, Garibaldi G, Bugarski-Kirola D, Berardo CG, Kapur S. Negative symptoms have greater impact on functioning than positive symptoms in schizophrenia: analysis of CATIE data. Schizophr Res 2012; 137: 147–50. 22316568 10.1016/j.schres.2012.01.015

[5] Strauss GP, Sandt AR, Catalano LT, Allen DN. Negative symptoms and depression predict lower psychological well-being in individuals with schizophrenia. Compr Psychiatry 2012; 53: 1137–44. 22770716 10.1016/j.comppsych.2012.05.009

[6] Pillny M, Schlier B, Lincoln TM. I just don't look forward to anything”. how anticipatory pleasure and negative beliefs contribute to goal-directed activity in patients with negative symptoms of psychosis. Schizophr Res 2020; 222: 429–36. 32389616 10.1016/j.schres.2020.03.059

[7] Leucht S, Corves C, Arbter D, Engel RR, Li C, Davis JM. Second-generation versus first-generation antipsychotic drugs for schizophrenia: a meta-analysis. Lancet 2009; 373: 31–41. 19058842 10.1016/S0140-6736(08)61764-X

[8] Sivec HJ, Montesano VL. Cognitive behavioral therapy for psychosis in clinical practice. Psychotherapy (Chic) 2012; 49: 258–70. 22642528 10.1037/a0028256

[9] Cassidy CM, Norman R, Manchanda R, Schmitz N, Malla A. Testing definitions of symptom remission in first-episode psychosis for prediction of functional outcome at 2 years. Schizophr Bull 2010; 36: 1001–8. 19321629 10.1093/schbul/sbp007 PMC2930352

[10] AlAqeel B, Margolese HC. Remission in schizophrenia: critical and systematic review. Harv Rev Psychiatry 2012; 20: 281–97. 23216066 10.3109/10673229.2012.747804

[11] Aleman A, Lincoln TM, Bruggeman R, Melle I, Arends J, Arango C, et al. Treatment of negative symptoms: Where do we stand, and where do we go? Schizophr Res 2017; 186: 55–62. 27293137 10.1016/j.schres.2016.05.015

[12] Möller H-J, Czobor P. Pharmacological treatment of negative symptoms in schizophrenia. Eur Arch Psychiatry Clin Neurosci 2015; 265: 567–78. 25895634 10.1007/s00406-015-0596-y

[13] Leucht S, Cipriani A, Spineli L, Mavridis D, Orey D, Richter F, et al. Comparative efficacy and tolerability of 15 antipsychotic drugs in schizophrenia: a multiple-treatments meta-analysis. Lancet 2013; 382: 951–62. 23810019 10.1016/S0140-6736(13)60733-3

[14] Deakin B, Suckling J, Barnes TRE, Byrne K, Chaudhry IB, Dazzan P, et al. The benefit of minocycline on negative symptoms of schizophrenia in patients with recent-onset psychosis (BeneMin): a randomised, double-blind, placebo-controlled trial. Lancet Psychiatry 2018; 5: 885–94. 30322824 10.1016/S2215-0366(18)30345-6 PMC6206257

[15] Klingberg S, Wölwer W, Engel C, Wittorf A, Herrlich J, Meisner C, et al. Negative symptoms of schizophrenia as primary target of cognitive behavioral therapy: results of the randomized clinical TONES study. Schizophr Bull 2011; 37(Suppl 2): S98–S110. 21860053 10.1093/schbul/sbr073 PMC3160126

[16] Velthorst E, Koeter M, van der Gaag M, Nieman DH, Fett AK, Smit F, et al. Adapted cognitive-behavioural therapy required for targeting negative symptoms in schizophrenia: meta-analysis and meta-regression. Psychol Med 2015; 45: 453–65. 24993642 10.1017/S0033291714001147

[17] Grant PM, Huh GA, Perivoliotis D, Stolar NM, Beck AT. Randomized trial to evaluate the efficacy of cognitive therapy for low-functioning patients with schizophrenia. Arch Gen Psychiatry 2012; 69: 121–7. 21969420 10.1001/archgenpsychiatry.2011.129

[18] Cella M, Bishara AJ, Medin E, Swan S, Reeder C, Wykes T. Identifying cognitive remediation change through computational modelling–effects on reinforcement learning in schizophrenia. Schizophr Bull 2014; 40: 1422–32. 24214932 10.1093/schbul/sbt152 PMC4193689

[19] Strauss GP, Waltz JA, Gold JM. A review of reward processing and motivational impairment in schizophrenia. Schizophr Bull 2014; 40(Suppl 2): S107–16. 24375459 10.1093/schbul/sbt197 PMC3934394

[20] Cella M, Stahl D, Morris S, Keefe RSE, Bell MD, Wykes T. Effects of cognitive remediation on negative symptoms dimensions: exploring the role of working memory. Psychol Med 2017; 47(15): 2593–601. 28866985 10.1017/S0033291717000757 PMC5647678

[21] Chien WT, Cheng HY, McMaster TW, Yip ALK, Wong JCL. Effectiveness of a mindfulness-based psychoeducation group programme for early-stage schizophrenia: an 18-month randomised controlled trial. Schizophr Res 2019; 212: 140–9. 31416744 10.1016/j.schres.2019.07.053

[22] Dean DJ, Bryan AD, Newberry R, Gupta T, Carol E, Mittal VA. A supervised exercise intervention for youth at risk for psychosis: an open-label pilot study. J Clin Psychiatry 2017; 78: e1167–e73. 29178684 10.4088/JCP.16m11365 PMC5995728

[23] Granholm E, Harvey PD. Social skills training for negative symptoms of schizophrenia. Schizophr Bull 2018; 44: 472–4. 29315427 10.1093/schbul/sbx184 PMC5890477

[24] Veerman SRT, Schulte PFJ, de Haan L. Treatment for negative symptoms in schizophrenia: a comprehensive review. Drugs 2017; 77: 1423–59. 28776162 10.1007/s40265-017-0789-y

[25] Lecomte T, Spidel A, Leclerc C, MacEwan GW, Greaves C, Bentall RP. Predictors and profiles of treatment non-adherence and engagement in services problems in early psychosis. Schizophr Res 2008; 102: 295–302. 18295458 10.1016/j.schres.2008.01.024

[26] Moher D, Liberati A, Tetzlaff J, Altman DG. Preferred reporting items for systematic reviews and meta-analyses: the PRISMA statement. Ann Intern Med 2009; 151: 264–9. 19622511 10.7326/0003-4819-151-4-200908180-00135

[27] Higgins JP, Thomas J, Chandler J, Cumpston M, Li T, Page MJ, et al. Cochrane Handbook for Systematic Reviews of Interventions. John Wiley & Sons, 2019. 10.1002/14651858.ED000142 PMC10284251 31643080

[28] Shea BJ, Bouter LM, Peterson J, Boers M, Andersson N, Ortiz Z, et al. External validation of a measurement tool to assess systematic reviews (AMSTAR). PLoS One 2007; 2: e1350. 18159233 10.1371/journal.pone.0001350 PMC2131785

[29] Richardson WS, Wilson MC, Nishikawa J, Hayward RS. The well-built clinical question: a key to evidence-based decisions. ACP J Club 1995; 123: A12–3. 10.7326/ACPJC-1995-123-3-A12 7582737

[30] Cramer H, Lauche R, Klose P, Langhorst J, Dobos G. Yoga for schizophrenia: a systematic review and meta-analysis. BMC Psychiatry 2013; 13: 32. 23327116 10.1186/1471-244X-13-32 PMC3608162

[31] Lutgens D, Gariepy G, Malla A. Psychological and psychosocial interventions for negative symptoms in psychosis: systematic review and meta-analysis. Br J Psychiatry 2017; 210: 324–32. 28302699 10.1192/bjp.bp.116.197103

[32] Sabe M, Sentissi O, Kaiser S. Meditation-based mind-body therapies for negative symptoms of schizophrenia: systematic review of randomized controlled trials and meta-analysis. Schizophr Res 2019; 212: 15–25. 31378557 10.1016/j.schres.2019.07.030

[33] Sabe M, Kaiser S, Sentissi O. Physical exercise for negative symptoms of schizophrenia: systematic review of randomized controlled trials and meta-analysis. Gen Hosp Psychiatry 2020; 62: 13–20. 31751931 10.1016/j.genhosppsych.2019.11.002

[34] Vogel JS, van der Gaag M, Slofstra C, Knegtering H, Bruins J, Castelein S. The effect of mind-body and aerobic exercise on negative symptoms in schizophrenia: a meta-analysis. Psychiatry Res 2019; 279: 295–305. 30879703 10.1016/j.psychres.2019.03.012

[35] Riehle M, Böhl MC, Pillny M, Lincoln TM. Efficacy of psychological treatments for patients with schizophrenia and relevant negative symptoms: a meta-analysis. Clin Psychol Eur 2020; 2: 1–23. 10.32872/cpe.v2i3.2899 PMC9645476 36398145

[36] Cella M, Preti A, Edwards C, Dow T, Wykes T. Cognitive remediation for negative symptoms of schizophrenia: a network meta-analysis. Clin Psychol Rev 2017; 52: 43–51. 27930934 10.1016/j.cpr.2016.11.009

[37] Burlingame GMS H, Hoppe L, Hunt I, Rosendahl J. Group therapy for schizophrenia: a meta-analysis. Psychother 2020; 57: 219–36. 10.1037/pst0000293 32478561

[38] Cramer H, Lauche R, Haller H, Langhorst J, Dobos G. Mindfulness- and acceptance-based interventions for psychosis: a systematic review and meta-analysis. Glob Adv Health Med 2016; 5: 30–43. 26937312 10.7453/gahmj.2015.083 PMC4756771

[39] Jansen JE, Gleeson J, Bendall S, Rice S, Alvarez-Jimenez M. Acceptance- and mindfulness-based interventions for persons with psychosis: a systematic review and meta-analysis. Schizophr Res 2020; 215: 25–37. 31780349 10.1016/j.schres.2019.11.016

[40] Jones C, Hacker D, Cormac I, Meaden A, Irving CB. Cognitive behaviour therapy versus other psychosocial treatments for schizophrenia. Cochrane Database Syst Rev 2012; 4: CD008712. 22513966 10.1002/14651858.CD008712.pub2 PMC4163968

[41] Orfanos S, Banks C, Priebe S. Are group psychotherapeutic treatments effective for patients with schizophrenia? A systematic review and meta-analysis. Psychother Psychosom 2015; 84: 241–9. 26022543 10.1159/000377705

[42] Polese D, Fornaro M, Palermo M, De Luca V, de Bartolomeis A. Treatment-resistant to antipsychotics: a resistance to everything? Psychotherapy in treatment-resistant schizophrenia and nonaffective psychosis: a 25-Year systematic review and exploratory meta-analysis. Front Psychiatr 2019; 10: 210. 10.3389/fpsyt.2019.00210 PMC6478792 31057434

[43] Turner DT, van der Gaag M, Karyotaki E, Cuijpers P. Psychological interventions for psychosis: a meta-analysis of comparative outcome studies. Am J Psychiatry 2014; 171: 523–38. 24525715 10.1176/appi.ajp.2013.13081159

[44] Jones C, Hacker D, Xia J, Meaden A, Irving CB, Zhao S, et al. Cognitive behavioural therapy plus standard care versus standard care for people with schizophrenia. Cochrane Database Syst Rev 2018; 12: CD007964. 30572373 10.1002/14651858.CD007964.pub2 PMC6517137

[45] Jones C, Hacker D, Meaden A, Cormac I, Irving CB, Xia J, et al. Cognitive behavioural therapy plus standard care versus standard care plus other psychosocial treatments for people with schizophrenia. Cochrane Database Syst Rev 2018; 11: Cd008712. 30480760 10.1002/14651858.CD008712.pub3 PMC6516879

[46] Firth J, Cotter J, Elliott R, French P, Yung AR. A systematic review and meta-analysis of exercise interventions in schizophrenia patients. Psychol Med 2015; 45: 1343–61. 25650668 10.1017/S0033291714003110

[47] Geretsegger M, Mössler KA, Bieleninik L, Chen XJ, Heldal TO, Gold C. Music therapy for people with schizophrenia and schizophrenia-like disorders. Cochrane Database Syst Rev 2017; 5: CD004025. 28553702 10.1002/14651858.CD004025.pub4 PMC6481900

[48] Jia R, Liang D, Yu J, Lu G, Wang Z, Wu Z, et al. The effectiveness of adjunct music therapy for patients with schizophrenia: a meta-analysis. Psychiatry Res 2020; 293: 113464. 33002835 10.1016/j.psychres.2020.113464

[49] Jauhar S, McKenna PJ, Radua J, Fung E, Salvador R, Laws KR. Cognitive-behavioural therapy for the symptoms of schizophrenia: systematic review and meta-analysis with examination of potential bias. Br J Psychiatry 2014; 204: 20–9. 24385461 10.1192/bjp.bp.112.116285

[50] Kurtz MM, Mueser KT. A meta-analysis of controlled research on social skills training for schizophrenia. J Consult Clin Psychol 2008; 76: 491–504. 18540742 10.1037/0022-006X.76.3.491

[51] Turner DT, McGlanaghy E, Cuijpers P, Van Der Gaag M, Karyotaki E, MacBeth A. A meta-analysis of social skills training and related interventions for psychosis. Schizophr Bull 2018; 44: 475–91. 29140460 10.1093/schbul/sbx146 PMC5890475

[52] Rodolico A, Bighelli I, Avanzato C, Concerto C, Cutrufelli P, Mineo L, et al. Family interventions for relapse prevention in schizophrenia: a systematic review and network meta-analysis. Lancet Psychiatry 2022; 9: 211–21. 35093198 10.1016/S2215-0366(21)00437-5

[53] Lejeune JA, Northrop A, Kurtz MM. A meta-analysis of cognitive remediation for schizophrenia: efficacy and the role of participant and treatment factors. Schizophr Bull 2021; 47: 997–1006. 33772310 10.1093/schbul/sbab022 PMC8266668

[54] Ma CF, Chan SKW, Chien WT, Bressington D, Mui EYW, Lee EHM, et al. Cognitive behavioural family intervention for people diagnosed with severe mental illness and their families: a systematic review and meta-analysis of randomized controlled trials. J Psychiatr Ment Health Nurs 2020; 27: 128–39. 31549461 10.1111/jpm.12567

[55] De Mare A, Cantarella M, Galeoto G. Effectiveness of integrated neurocognitive therapy on cognitive impairment and functional outcome for schizophrenia outpatients. Schizophr Res Treat 2018; 2018: 2360697. 10.1155/2018/2360697 PMC6215544 30420918

[56] Kay SR, Fiszbein A, Opler LA. Positive and Negative Syndrome Scale (PANSS). MHS, 2012. 10.1093/schbul/13.2.261 3616518

[57] Andreasen NC. The scale for the assessment of negative symptoms (SANS): conceptual and theoretical foundations. Br J Psychiatry 1989; 155(S7): 49–52. 2695141

[58] Velligan D, Prihoda T, Dennehy E, Biggs M, Shores-Wilson K, Crismon ML, et al. Brief psychiatric rating scale expanded version: how do new items affect factor structure? Psychiatry Res 2005; 135: 217–28. 15993949 10.1016/j.psychres.2005.05.001

[59] Derogatis LR, Spencer P. Brief Symptom Inventory: BSI. Pearson Upper Saddle River, 1993.

[60] Alphs L, Morlock R, Coon C, van Willigenburg A, Panagides J. The 4-item negative symptom assessment (NSA-4) instrument: a simple tool for evaluating negative symptoms in schizophrenia following brief training. Psychiatry (Edgmont) 2010; 7: 26–32. 20805916 PMC2922363

[61] Kirkpatrick B, Strauss GP, Nguyen L, Fischer BA, Daniel DG, Cienfuegos A, et al. The brief negative symptom scale: psychometric properties. Schizophr Bull 2011; 37: 300–5. 20558531 10.1093/schbul/sbq059 PMC3044634

[62] Asberg M, Schalling D. Construction of a new psychiatric rating instrument, the comprehensive psychopathological rating scale (CPRS). Prog Neuropsychopharmacol 1979; 3: 405–12. 400996 10.1016/0364-7722(79)90055-9

[63] Wykes T, Steel C, Everitt B, Tarrier N. Cognitive behavior therapy for schizophrenia: effect sizes, clinical models, and methodological rigor. Schizophr Bull 2008; 34: 523–37. 17962231 10.1093/schbul/sbm114 PMC2632426

[64] Sarin F, Wallin L, Widerlov B. Cognitive behavior therapy for schizophrenia: a meta-analytical review of randomized controlled trials. Nord J Psychiatry 2011; 65: 162–74. 21563994 10.3109/08039488.2011.577188

[65] Hodann-Caudevilla RM, Díaz-Silveira C, Burgos-Julián FA, Santed MA. Mindfulness-based interventions for people with schizophrenia: a systematic review and meta-analysis. Int J Environ Res Public Health 2020; 17(13): 4690. 32629764 10.3390/ijerph17134690 PMC7369977

[66] Tonarelli SB, Pasillas R, Alvarado L, Dwivedi A, Cancellare A. Acceptance and commitment therapy compared to treatment as usual in psychosis: a systematic review and meta-analysis. Afr J Psychiatry 2016; 19: 206–11.

[67] Liu YC, Li IL, Hsiao FH. Effectiveness of mindfulness-based intervention on psychotic symptoms for patients with schizophrenia: a meta-analysis of randomized controlled trials. J Adv Nurs 2021; 77: 2565–80. 33450107 10.1111/jan.14750

[68] Laws KR, Darlington N, Kondel TK, McKenna PJ, Jauhar S. Cognitive behavioural therapy for schizophrenia - outcomes for functioning, distress and quality of life: a meta-analysis. BMC Psychol 2018; 6: 32. 30016999 10.1186/s40359-018-0243-2 PMC6050679

[69] Nenke MA, Hahn LA, Thompson CH, Liu D, Galletly CA. Psychosis and cardiovascular disease: is diet the missing link? Schizophr Res 2015; 161: 465–70. 25560938 10.1016/j.schres.2014.12.012

[70] Lally J, Spaducci G, Gardner-Sood P, Atakan Z, Greenwood K, Di Forti M, et al. Tobacco smoking and nicotine dependence in first episode and established psychosis. Asian J Psychiatr 2019; 43: 125–31. 31132542 10.1016/j.ajp.2019.05.002

[71] Martland R, Teasdale S, Murray RM, Gardner-Sood P, Smith S, Ismail K, et al. Dietary intake, physical activity and sedentary behaviour patterns in a sample with established psychosis and associations with mental health symptomatology. Psychol Med 2021; 23: 1–11. 10.1017/S0033291721003147 PMC10009388 34420532

[72] Laska KM, Gurman AS, Wampold BE. Expanding the lens of evidence-based practice in psychotherapy: a common factors perspective. Psychotherapy 2014; 51: 467–81. 24377408 10.1037/a0034332

[73] Lincoln TM, Suttner C, Nestoriuc Y. Wirksamkeit kognitiver Interventionen für Schizophrenie. [Effectiveness of cognitive interventions for schizophrenia.] Psychol Rundschau 2008; 59: 217–32.

[74] Edwards CJ, Cella M, Emsley R, Tarrier N, Wykes THM. Exploring the relationship between the anticipation and experience of pleasure in people with schizophrenia: an experience sampling study. Schizophr Res 2018; 202: 72–9. 30007868 10.1016/j.schres.2018.06.040 PMC6294730

[75] Miller R, Hollist C. Attrition bias. In Encyclopaedia of Measurement and Statistics (ed N Salkind): Vol. 1, 57–60. Sage Publications, Inc., 2007.

[76] Blanchard JJ, Cohen AS. The structure of negative symptoms within schizophrenia: implications for assessment. Schizophr Bull 2006; 32: 238–45. 16254064 10.1093/schbul/sbj013 PMC2632211

[77] Strauss GP, Hong LE, Gold JM, Buchanan RW, McMahon RP, Keller WR, et al. Factor structure of the Brief Negative Symptom Scale. Schizophr Res 2012; 142: 96–8. 23062750 10.1016/j.schres.2012.09.007 PMC3502636

[78] Strauss GP, Nuñez A, Ahmed AO, Barchard KA, Granholm E, Kirkpatrick B, et al. The latent structure of negative symptoms in schizophrenia. JAMA Psychiatry 2018; 75: 1271–9. 30208377 10.1001/jamapsychiatry.2018.2475 PMC6583036

[79] Kring AM, Gur RE, Blanchard JJ, Horan WP, Reise SP. The Clinical Assessment Interview for Negative Symptoms (CAINS): final development and validation. Am J Psychiatry 2013; 170: 165–72. 23377637 10.1176/appi.ajp.2012.12010109 PMC3785242

[80] Kirkpatrick B, Saoud JB, Strauss GP, Ahmed AO, Tatsumi K, Opler M, et al. The Brief Negative Symptom Scale (BNSS): sensitivity to treatment effects. Schizophr Res 2018; 197: 269–73. 29275856 10.1016/j.schres.2017.11.031

[81] Galderisi S, Mucci A, Dollfus S, Nordentoft M, Falkai P, Kaiser S, et al. EPA guidance on assessment of negative symptoms in schizophrenia. Eur Psychiatry 2021; 64: e23. 33597064 10.1192/j.eurpsy.2021.11 PMC8080207

[82] Smith V, Devane D, Begley CM, Clarke M. Methodology in conducting a systematic review of systematic reviews of healthcare interventions. BMC Med Res Methodol 2011; 11: 15. 21291558 10.1186/1471-2288-11-15 PMC3039637

[83] Higgins JPT, Altman DG, Gøtzsche PC, Jüni P, Moher D, Oxman AD, et al. The Cochrane collaboration's tool for assessing risk of bias in randomised trials. BMJ 2011; 343: d5928. 22008217 10.1136/bmj.d5928 PMC3196245

[84] Lincoln TM, Riehle M, Pillny M, Helbig-Lang S, Fladung AK, Hartmann-Riemer M, et al. Using functional analysis as a framework to guide individualized treatment for negative symptoms. Front Psychol 2017; 8: 2108. 29259567 10.3389/fpsyg.2017.02108 PMC5723417

[85] Cella M, Tomlin P, Robotham D, Green P, Griffiths H, Stahl D, et al. Virtual reality therapy for the negative symptoms of schizophrenia (V-NeST): a pilot randomised feasibility trial. Schizophr Res 2022; 248: 50–7. 35939920 10.1016/j.schres.2022.07.013

