# Supplementary material for: Psychosocial and behavioural interventions for the negative symptoms of schizophrenia: a systematic review of efficacy meta-analyses
Source: Br J Psychiatry. 2023 Jul;223(1):321–31. doi: 10.1192/bjp.2023.21 (PMC10331321; doi:10.1192/bjp.2023.21)
Supplement: Supplementary file 1 [file S0007125023000211sup001.zip › S0007125023000211sup001.docx]

Appendix A: Search strategy

| **#** | **Searches** | **Results** |  |
| --- | --- | --- | --- |
| 1 | Psychotic or Schizophrenia | 202027 |  |
| 2 | delusions.mp. or exp delusion | 37285 |  |
| 3 | cognition disorders.mp. or exp cognitive defect | 572528 |  |
| 4 | schizophrenia/ or parapsychology/ or cognition/ or delusion/ or psychosis/ | 544859 |  |
| 5 | 1 or 2 or 3 or 4 | 1026037 |  |
| 6 | negative symptoms.mp. or exp negative syndrome/ | 19506 |  |
| 7 | alogia.mp. | 437 |  |
| 8 | speech poverty.mp | 8 |  |
| 9 | poverty of speech.mp. | 171 |  |
| 10 | anhedonia/ or anhedon*.mp. | 10360 |  |
| 11 | deficit syndrome.mp. or exp negative syndrome | 13274 |  |
| 12 |  |  |  |
| 13 | motivation difficult*.mp. | 23 |  |
| 14 | amotivation.mp. | 671 |  |
| 15 | poor motivation.mp. | 305 |  |
| 16 | avolition*.mp. | 803 |  |
| 17 | apath*.mp. | 16539 |  |
| 18 | affective blunting.mp. | 92 |  |
| 19 | emotional blunting.mp. | 310 |  |
| 20 | blunt* affect.mp. | 1270 |  |
| 21 | affective flat*.mp. | 299 |  |
| 22 |  |  |  |
| 23 | flat affect.mp. or exp blunted affect | 1106 |  |
| 24 | 6 or 7 or 8 or 9 or 10 or 11 or 12 or 13 or 14 or 15 or 16 or 17 or 18 or 19 or 20 or 21 or 22 or 23 | 46357 |  |
| 25 | exp psychotherapy | 299905 |  |
| 26 | psychotherap*.mp. | 133312 |  |
| 27 | psychosocial therap*.mp. | 656 |  |
| 28 | Psychosocial intervention*.mp. | 9887 |  |
| 29 | cognitive behavioural therapy.mp. or exp cognitive behavioral therapy/ | 25925 |  |
| 30 | cognitive behavio?r* therap*.mp. | 38720 |  |
| 31 | cognitive behavio*r intervention.mp. | 39 |  |
| 32 | Cognitive therap*.mp. | 45603 |  |
| 33 | cognitive intervention*.mp. | 1873 |  |
| 34 | negative symptom* intervention*.mp. | 7 |  |
| 35 | negative symptom* therap*.mp. | 1 |  |
| 36 | (Acceptance and commitment).mp. | 3371 |  |
| 37 | exp mindfulness | 12897 |  |
| 38 | Mindfulness.mp. | 27 |  |
| 39 | positive psychology.mp. or exp positive psychology/ | 1740 |  |
| 40 | compassion focussed therap*.mp. | 10 |  |
| 41 | cognitive remediation.mp. or exp cognitive remediation therapy | 2787 |  |
| 42 | behaviour therapy.mp. or exp behavior therapy/ | 71230 |  |
| 43 | behavio?r therap*.mp. | 54567 |  |
| 44 | exp rehabilitation/ or rehabilitation.mp | 709061 |  |
| 45 | exercise.mp. or exp exercise/ | 614746 |  |
| 46 | narrative therapy.mp. or exp narrative therapy | 645 |  |
| 47 | 25 or 26 or 27 or 28 or 29 or 30 or 31 or 32 or 33 or 34 or 35 or 36 or 37 or 38 or 39 or 40 or 41 or 42 or 43 or 44 or 45 or 46 | 1523669 |  |
| 48 | 5 and 24 | 473760 |  |
| 49 | limit 48 to (english language and (meta analysis or "systematic review")) | 693 |  |
