# Supplementary material for: Psychosocial and behavioural interventions for the negative symptoms of schizophrenia: a systematic review of efficacy meta-analyses
Source: Br J Psychiatry. 2023 Jul;223(1):321–31. doi: 10.1192/bjp.2023.21 (PMC10331321; doi:10.1192/bjp.2023.21)
Supplement: Supplementary file 1 [file S0007125023000211sup001.zip › S0007125023000211sup002.docx]

| Supplementary Appendix 2  *Drop-out rates reported information* | |
| --- | --- |
| Review, year | **Information on drop-out rate available (Y/N)** |
| Burlingame et al., 2020 | Y |
| Cella et al., 2017 | Y |
| Cramer et al., 2013 | N* |
| Cramer et al., 2016 | N |
| De mare et al., 2018 | Y |
| Firth et al., 2015 | Y |
| Geretsegger et al., 2017 | N |
| Hodann-Caudevilla et al., 2020 | Y |
| Jansen et al., 2020 | N |
| Jauhar et al., 2014 | N |
| Jia et al., 2020 | N |
| Jones et al., 2018a | Y |
| Jones et al., 2018b | N |
| Kurtz et al., 2008 | N |
| Liu et al., 2021 | N** |
| Lejeune et al., 2021 | N |
| Lutgens et al., 2017 | N*** |
| Ma et al., 2020 | N |
| Orfanos et al., 2015 | N |
| Polese et al., 2015 | N**** |
| Riehle et al., 2020 | Y |
| Rodolico et al., 2022 | Y |
| Sabe et al., 2019 | N |
| Sabe et al., 2020 | N |
| Sarin et al., 2011 | Y |
| Tonarelli et al., 2016 | N |
| Turner 2018 | N |
| Turner, 2014 | N |
| Velthorst et al., 2015 | N |
| Vogel et al., 2019 | Y |
| Wykes et al., 2008 | N |

** None of the studies included in the review had acceptable dropout rates or applied an intention to treat analysis; ** Report retention rates; *** Most of the studies included did not account for attrition; ****Majority of the studies included did not report accurate dropout rates.*
