# Supplementary material for: Psychosocial and behavioural interventions for the negative symptoms of schizophrenia: a systematic review of efficacy meta-analyses
Source: Br J Psychiatry. 2023 Jul;223(1):321–31. doi: 10.1192/bjp.2023.21 (PMC10331321; doi:10.1192/bjp.2023.21)
Supplement: Supplementary file 1 [file S0007125023000211sup001.zip › S0007125023000211sup003.docx]

Appendix C: AMSTAR Quality Ratings

*(Y= Yes; N=No; PY=Partially Yes)*

|  | | |  |  |  |  |  |  |  |  |  |  |  |  |  |  |  |
| --- | --- | --- | --- | --- | --- | --- | --- | --- | --- | --- | --- | --- | --- | --- | --- | --- | --- |
| Review, Year | Item 1 | Item 2 | Item 3 | Item 4 | Item 5 | Item 6 | Item 7 | Item 8 | Item 9 | Item 10 | Item 11 | Item 12 | Item 13 | Item 14 | Item 15 | Item 16 | AMSTAR Rating of Review Quality |
| Rodolico et al., 2022 | Y | Y | N | PY | Y | Y | N | Y | Y | N | N | Y | N | N | Y | Y | Critically low Quality |
| Liu et al., 2021 | Y | N | N | PY | Y | Y | N | Y | Y | N | Y | Y | Y | Y | N | Y | Critically low Quality |
| Lejeune et al., 2020 | Y | N | N | PY | N | Y | N | PY | PY | N | Y | Y | Y | N | N | Y | Critically low Quality |
| Burlingame et al., 2020 | Y | N | Y | PY | N | Y | N | Y | Y | N | Y | Y | N | Y | Y | N | Critically low Quality |
| Hodann-Caudevilla et al., 2020 | Y | PY | N | N | Y | N | N | PY | Y | N | Y | Y | Y | Y | Y | Y | Critically low Quality |
| Jansen et al., 2020 | Y | PY | N | Y | Y | Y | N | Y | Y | N | Y | Y | N | Y | Y | N | Critically low Quality |
| Jia et al., 2020 | Y | N | N | PY | Y | Y | N | PY | PY | N | Y | Y | N | Y | Y | Y | Critically low Quality |
| Ma et al., 2020 | Y | Y | N | Y | Y | N | N | Y | Y | N | Y | Y | Y | N | Y | Y | Low Quality |
| Riehle et al., 2020 | Y | N | N | N | N | N | N | PY | N | N | Y | Y | N | Y | Y | Y | Critically low Quality |
| Sabe et al., 2020 | Y | N | N | Y | N | N | N | PY | Y | N | Y | Y | Y | Y | Y | Y | Low Quality |
| Vogel et al., 2019 | Y | N | N | Y | Y | Y | N | Y | Y | N | Y | Y | Y | Y | Y | N | Low Quality |
| Sabe et al., 2019 | Y | N | N | Y | Y | Y | Y | Y | Y | Y | Y | Y | Y | Y | N | Y | Low Quality |
| De Mare et al., 2018 | N | N | N | PY | Y | N | N | PY | Y | N | Y | N | N | Y | N | Y | Critically low Quality |
| Jones et al., 2018a | Y | Y | N | Y | Y | Y | Y | Y | Y | Y | Y | Y | Y | Y | N | Y | Low Quality |
| Jones et al., 2018b | Y | PY | N | Y | Y | Y | Y | Y | Y | Y | Y | Y | Y | Y | N | Y | Low Quality |
| Turner 2018 | Y | N | N | Y | N | Y | N | PY | PY | N | Y | Y | Y | Y | Y | Y | Critically low Quality |
| Cella et al., 2017 | Y | PY | N | Y | Y | Y | N | Y | PY | N | Y | Y | Y | Y | Y | N | Low quality |
| Geretsegger et al., 2017 | Y | PY | N | Y | Y | Y | Y | Y | Y | N | Y | Y | Y | Y | Y | Y | Moderate Quality |
| Lutgens et al., 2017 | Y | N | N | Y | N | Y | N | PY | Y | N | Y | Y | Y | N | Y | Y | Critically low Quality |
| Cramer et al., 2016 | Y | N | N | PY | Y | Y | N | Y | Y | N | Y | Y | Y | Y | Y | Y | Critically low Quality |
| Tonarelli et al., 2016 | N | N | N | Y | Y | Y | N | N | N | N | Y | N | N | Y | N | N | Critically low Quality |
| Firth et al., 2015 | Y | N | Y | PY | Y | Y | Y | PY | Y | N | Y | Y | N | N | Y | Y | Critically low Quality |
| Orfanos et al., 2015 | Y | N | N | Y | Y | Y | N | PY | Y | N | Y | Y | Y | N | Y | Y | Critically low Quality |
| Polese et al., 2015 | N | N | N | PY | Y | Y | N | Y | Y | Y | Y | N | N | N | Y | Y | Critically low Quality |
| Velthorst et al., 2015 | Y | N | N | Y | N | N | N | Y | Y | N | Y | Y | Y | Y | Y | Y | Critically low Quality |
| Jauhar et al., 2014 | Y | N | N | Y | N | Y | Y | PY | PY | N | Y | Y | Y | N | Y | Y | Low Quality |
| Turner, 2014 | N | N | N | PY | Y | Y | N | PY | Y | N | Y | Y | Y | Y | Y | Y | Critically low Quality |
| Cramer et al., 2013 | Y | N | N | Y | Y | Y | N | Y | Y | N | Y | Y | Y | Y | Y | Y | Critically low Quality |
| Sarin et al., 2011 | Y | N | N | PY | Y | Y | N | Y | Y | N | Y | Y | Y | N | N | Y | Critically low Quality |
| Kurtz & Mueser 2008 | N | N | N | PY | N | N | N | Y | N | N | Y | Y | N | Y | Y | N | Critically low Quality |
| Wykes et al., 2008 | Y | N | N | Y | Y | Y | N | PY | Y | N | Y | Y | Y | Y | Y | N | Critically low Quality |
