# Supplementary material for: Psychosocial and behavioural interventions for the negative symptoms of schizophrenia: a systematic review of efficacy meta-analyses
Source: Br J Psychiatry. 2023 Jul;223(1):321–31. doi: 10.1192/bjp.2023.21 (PMC10331321; doi:10.1192/bjp.2023.21)
Supplement: Supplementary file 1 [file S0007125023000211sup001.zip › S0007125023000211sup004.docx]

**Calculating Standardized Mead Differences from Mean Differences**

When results were reported as a mean difference with a confidence interval, we calculated the standardized mean difference by deriving the standard deviation from the width of the confidence interval according to this formula:

SD = $\sqrt{n}$ x (upper limit – lower limit) / 3.92

(See section 6.5.2.2 of the Version 6.3, 2022 of the Cochrane Handbook for Systematic Reviews of Interventions: <https://training.cochrane.org/handbook/current/chapter-06>).
