# Supplementary material for: Psychosocial and behavioural interventions for the negative symptoms of schizophrenia: a systematic review of efficacy meta-analyses
Source: Br J Psychiatry. 2023 Jul;223(1):321–31. doi: 10.1192/bjp.2023.21 (PMC10331321; doi:10.1192/bjp.2023.21)
Supplement: Supplementary file 1 [file S0007125023000211sup001.zip › S0007125023000211sup005.docx]

Supplementary Table 1 Review characteristics

| Review Year Intervention | Inclusion Criteria | N Studies Included | Population | N Participants | Age Range (yrs)a | Intervention | Length of Intervention (on average) | Control Group | Measure(s) of NS | Other Outcome Measures Included | Statistical Analysis | Results |
| --- | --- | --- | --- | --- | --- | --- | --- | --- | --- | --- | --- | --- |
| Kurtz et al 2008 | RCTs; SST intervention, majority of sample with a diagnosis of schizophrenia or schizoaffective disorder. | 6 (studies included for NS only) | Schizophrenia or schizoaffective disorder. | 363 | 37.7 | SST needed to include the following core behavioural techniques: instructions on the skill, live or taped modelling or role-play rehearsal, positive & corrective feedback. | Ranged between a minimum of 8 h to 312 h of training, occurring over 2-104 weeks. | Active interventions or TAU. | PANSS, SANS, BPRS. | Content mastery of skills taught, social and daily living skills, community and institutional functioning, relapse. | Cohen’s d. | Moderate mean effect size for SST (d = 0.40, P < 0.05) that was unstable. |
| Wykes et al 2008 | RCT’s, majority of cohort with SCZ, In the experimental group CBT was an adjunct to TAU, CBT treatment was targeted at one of following outcomes (positive or NS, functioning, mood, hopelessness/suicidality, social anxiety). | 23 (studies included for NS only) | Majority of participants had to have a diagnosis of schizophrenia. | 1268 (overall) | 58.2 (overall) | CBT-p | − | TAU | PANSS | Also investigated outcomes on target symptoms, positive symptoms, functioning, mood and hopelessness | Mean weighted effect size. | An effect of CBT-p on NS was found (Mean weighted effect size = 0.437, P < 0.05). |
| Sarin et al 2011, Psychological Therapy | RCTs with a low risk of bias. Quasi-experimental studies were excluded. Published studies. Participants had to be over 18 years old. Studies with less than 10 participants were excluded. | 22 (overall) NS studies included for post-treatment = 3 NS studies included for follow-up = 3 | People schizophrenia, schizoaffective syndrome, or delusion disorder), diagnosed by ICD-10 or DSM-5. | 2469 (overall) Studies for NS post-treatment: N = 328 Studies for NS at follow-up: N = 318 | 36 | CBT interventions that gave a description of how the therapy was completed. | − | TAU | PANSS, PSYRATS, SANS | BDI-II, Beck Anxiety Inventory, Comprehensive psychopathological rating scale, use of medication and relapse. | Hedges’ g | Small significant effect favouring CBT at follow-up 3–15 months after treatment (SMD = −0.21, P = 0.03) when CBT was compared to other psychological treatments. No significant effects post-treatment or when CBT was compared to TAU. |
| Cramer et al, 2013, Exercise | RCTs and randomized cross-over studie using standardised measures of NS. | 5 (3 of which looked at NS) | DSM, RDC, ICD SCZ or treated for SCZ, included comorbid physical or mental disorders. | 198 | 28.2−48.1 | Yoga interventions including at least 1: physical activity, breath control, meditation, yoga based lifestyle advice | Ranged between a single 30 min session to 4 months. | Exercise or usual care. | PANSS | Cognitive function, Socio-Occupational Functioning Scale (SOFS), hospitalisation, safety of intervention. | SMD | No evidence for short-term effects of yoga on NS compared to TAU (SMD = −0.59, 95% CI = −1.87 to 0.69; P = 0.36, I squared = 80%). No evidence for short-term effects of yoga on NS compared to exercise (SMD = -0.28, P = 0.63) |
| Jauhar et al 2014, Psychological Therapy | RCT. Majority of patients to have a diagnosis of schizophrenia, schizoaffective or non-affective functional psychosis. Studies to include a parallel control group. Individual and group CBT included. | 34 (studies included for NS only) | Diagnosis of schizophrenia, schizoaffective or non-affective functional psychosis, either made clinically or according to diagnostic criteria. | − | − | CBT | − | Waitlist, TAU or an intervention designed to control for the non-specific effects of therapy (e.g. recreation and support, group support, befriending) | PANSS, SANS, BPRS negative factor, NS scale derived from the CPRS | Positive symptoms, Hallucinations (PSYRATS, BPRS, CPRS). | Hedge's g. | Significant beneficial effect of CBT for NS (g = −0.13, P = 0.03). |
| Turner et al 2014, Psychological Therapy | RCTs, studies included comparison of at least two psychological interventions intended to be therapeutic for psychosis. Trials were excluded if the comparison condition could not be deemed an active psychological intervention. | 48 (overall) | Primarily patients with diagnoses of psychotic disorders. | 3295 participants (total) | − | Befriending, CBT, CR, psychoeducation, SST, supportive counselling. | − | Active psychological intervention | PANSS, BPRS, SANS, PSYRATS | − | Effect sizes, Hedge’s g. | SST was more efficacious compared with other interventions pooled for NS (g = 0.267, P < 0.05). No evidence to suggests any of the other interventions were more efficacious. |
| Firth et al, 2015, Exercise | Studies reporting effect of exercise on ≥ quantitative measure of physical/ mental health. Interventions that only used yoga, muscular relaxation or adventure activities were excluded. Published in English after 1994. | 20 (5 of which looked at NS) | Any non-affective psychotic disorder. No restrictions placed on the severity or duration of illness. | 659 | 25–52 | ‘Exercise’ defined as any structured and repetitive physical activity with objective of improving or maintaining physical fitness, eg. Jogging, cycying | − | Waitlist, TAU, non-specific intervention, Occupational therapy (n = 1) | SANS, PANSS | Physical health, physical fitness, functioning and disability, comorbid mental health disorders, brain structure and neurocognitive functioning. | SMD | Four trials that implemented moderate to vigorous exercise for an average of 90 min per week showed a reduction in NS, with pooled SMDs of−0.44 (95% CI −0.78 to −0.09). |
| Orfanos et al 2015, Psychological Therapy | RCTs, included participants with a diagnosis of schizophrenia and related disorders. Studies excluded if they involved individualised treatment, involved family therapy and/or family intervention, and included participants aged ≤ 16 years. | 27 (studies included for NS only) | A sample with >85% of participants diagnosed with schizophrenia, schizotypal, schizoaffective and/or other non-affective psychotic disorders outlined in DSM. | Treatment Vs TAU = 893 Treatment Vs Active Sham = 783 | 17–78 (overall) | Non-specific group therapy: non-verbal arts therapies (music, body-oriented psychotherapy and art therapy), CR, psychoeducation and integrated approaches. | − | TAU or active sham. | PANSS | Positive symptoms and social functioning | SMD | Significant difference between intervention group and TAU (SMD = –0.37, P < 0.00), but not active sham. |
| Velthorst et al 2015, Psychological Therapy | RCTs, studies that reported on NS as outcomes; CBT was targeted at one of the following outcome domains: psychotic symptomatology, NS, (social) functioning, self-esteem or cannabis use, Published in English | 28 (NS as secondary outcome) 2 (NS as primary outcome) | Chronic or mixed patient group and recent onset. | 2312 | 21–54 | CBT | − | TAU, enriched TAU condition (may also include psychoeducation, social skills training, befriending, enhanced supportive therapy, CR, goal focussed supportive contact. | PANSS |  | Hedge’s g | No significant effects for NS when NS as primary or secondary outcome. |
| Tonarelli et al., 2016, Psychological Therapy | RCTs. Intervention delivered in an individual format, published in English, diagnosis of psychosis, schizophrenia, or major depression with psychosis. | 2 (studies included for NS only) | Adults with a diagnosis of schizophrenia, psychosis, or major depressive disorder with psychosis | 67 (NS analysis only) | − | ACT delivered in an individual format. | − | TAU defined as medical treatment and group psychotherapy or individual psychoeducation. | PANSS | Hospitalisation | SMD | Significant effects for NS (SMD = 0.648, P = 0.008) |
| Cramer et al 2016, Psychological Therapy | RCTs, adults with psychotic disorders, included comorbid physical or mental disorders, Primary outcome included improvement in the severity of symptoms of psychosis or hospitalization^^No language restrictions were applied. | 3 (studies included for NS only) | Adults with psychotic disorders diagnosed by manual, any other clinician-based diagnosis criterion; or unclear criteria but receiving treatment | 111 (NS analysis only) | 25.6-41.6 | ACT or cceptance-based behaviour therapy.  Mindfulness-based Cognitive Therapy (MBCT) or Mindfulness-based Stress Reduction (MBSR) | − | TAU, attention control, or other active non-pharmacological interventions | PANSS, BPSR, CGI or nay other validated scale. | Hospitalisation, affect, acceptance, mindfulness, safety of the intervention. | SMD | No significant difference between acceptance-based interventions and control (p = 0.23) |
| Geretsegger et al, 2017, Music | RCTs music therapy vs standard care, placebo therapy, or no treatment. Excluded data where >30% in any group were lost to follow-up. | 18 (5 of which looked at NS) | SCZ or SCZ-like disorders diagnosed by any criteria,. | 1215 in total, 177 for NS analysis. | − | Music therapy: individual, group music appreciation & singing, active and receptive group music therapy. | Ranged from 24-78 sessions. | Standard care | SANS | Global state, depression, social functioning, cognitive functioning, patient behaviour, patient satisfaction, general QoL | SMD | Significant beneficial effects for music therapy on NS (SMD −0.55 P < 0.00). But low quality evidence. |
| Cella et al, 2017, Psychological Therapy | RCTs, with appropriate comparison group (TAU or another active treatment), > 75% with SCZ / schizoaffective disorder, assessed NS using a validated measure. | 45 | Diagnosis of SCZ or schizoaffective disorder, | 2511 | M = 35.1 | Course of CR incl massed practice, errorless learning & scaffolding. Primary target had to be ≥ 1 cognitive domains. | − |  | PANSS, BPRS, SANS | − | Hedge’s g. | CR was effective in reducing levels of NS at post-treatment (RE: g = −0.34, P < 0.01; FE: g = −0.35, P < 0.01). Effectiveness of CR on NS was maintained at follow- up. |
| Lutgens et al 2017, Psychological Therapy | RCTs investigating a psychological or psychosocial intervention, outcomes using a valid and reliable NS scale, a majority a SCZ spectrum or other non-organic psychotic disorder. | 72 | Diagnosis of SCZ spectrum or other non-organic psychotic disorder. | − | − | CBT, Skills training, OT & cognitive adaptation training; neurocognitive therapies; PE; art & music therapies; family based interventions; | CBT: M = 28 sessions, over a mean of 6 months.  SKILLS TRAINING: M = 25.5 sessions, mean of 7 monthss NEUROCOGNITIVE TRAINING: M = 42 sessions, mean of 3.75 months  PE: M = 26.7 sessions, mean of 3 months ARTS AND MUSIC: M = 22 sessions, mean of 4.25 months. FAMILY: M = 23 sessions, mean of 10.9 months. | TAU or active control. | SANS, PANSS | − | SMD | CBT: Effective intervention for NS (SMD = −0.34, P = 0.00).  SKILL TRAINING: Intervention significantly reduced overall NS (pooled SMD = 0.44, P = 0.00). NEUROCOGNITIVE TRAINING: no overall effect on NS. EXERCISE: An effect on NS was found (SMD = -0.36, P = 0.04) ARTS AND MUSIC: No overall effect on NS. FAMILY: No effect on NS. MISCELLANEOUS: A significant effect of all miscellaneous studies was found (SMD = −0.42, 95% CI = −0.77 to −0.07) |
| De Mare et al 2018, Psychological Therapy | RCTs: Integrated Neurocognitive Therapy (INT) vs TAU, aged ≥18 years | 2 (studies included for NS only) | Diagnosis of SCZ or schizoaffective disorder. | 217 (NS analysis) | M = 34.9 | 30 biweekly sessions of INT with each session lasting 90 min | 30 weeks | TAU | PANSS | GAF | Mean difference | Significant effects for NS after 15 weeks (MD −2.99, P < 0.0001) and at 9–12 month follow-up (MD −2.47, P = 0.003). |
| Jones et al, 2018b, Psychological Therapy | RCTs : CBT or any psychosocial care, trials where >50% of participants had diagnosis of SCZ | 36 (total)  11 reporting on NS only. | Diagnosis of SCZ diagnosed by any criteria, | 3542 people with SCZ | 18–65 | CBT Other psychosocial interventions incl standard care – eg supportive therapy, psychoeducation, family therapy | − | Psychosocial treatments. | PANSS, SANS | Global state, mental state, adverse effects or events, functioning, QoL, satisfaction, engagement, economic costs. |  | No difference between CBT & non-active psychosocial therapy at short term, medium term and long term. No clear difference between CBT & active psychosocial therapy at short or medium term, but a clear effect favouring CBT at long term. (MD −1.34, 95% CI −2.57 to −0.11). |
| Jones et al., 2018a, Psychological Therapy | RCTs comparing CBT + TAU vs. TAU alone, at ≥50% people with SCZ or related disorders as defined by any criteria | 60 (25 in NS analyses) | ≥50% people with SCZ or related disorders as defined by any criteria | 231–1436 (included in NS analyses) | 16–78 | CBT + TAU | Short-term: < 12 weeks Medium-term: 13-26 weeks Long-term: > 26 weeks | TAU | PANSS, SANS | Relapse, hospitalisation, Mental state, adverse effects, functioning, QoL Satisfaction, engagement, economic costs | MD, fixed & random effects model | PANSS: moderate short-term (MD = −3.35, P < 0.001), small medium- (MD = −1.47, P < 0.001) and long-term effects (MD = −1.47, P < 0.001) of CBT reducing NS. SANS: no evidence of CBT reducing NS (short-term: MD = −4.11, P = 0.20). |
| Turner et al, 2018, Psychological Therapy | RCTs, psychosis population SST intervention, relevant measures to assess outcomes. | 17 | All psychotic disorders | 1437 (total) | − | Intervention was defined as SST or was primarily intended to improve social performance | − | TAU Active controls - Supportive counselling, psychoeducation, CR, CBT, control skills training, family intervention, supportive counselling. | PANSS, BPRS, BNSS, BSI, NSA-16 | General symptoms and social performance | Hedge’s g. Q and I squared for heterogeneity. | SST more efficacious for NS when compared to all comparators pooled (g = 0.19, P = 0.01) and TAU (g = 0.31, P = 0.01), but not Active controls (g = 0.14, P = 0.20) SST did not demonstrate superiority against Supportive Counselling for NS, but this comparison was underpowered. |
| Sabe et al, 2019, Exercise | Rater-blind RCTs. Studies that compare MBE to a non-specific control intervention. Exercise ≥30 min. Intervention duration ≥3 wks | 15 | SCZ, schizoaffective disorder, & 1study included psychotic disorders. Majority outpatients, 3 studies inpatients. | 1081 | 18–65 | Yoga (n = 10), Tai chi (n = 2), Mindfulness (n = 3) | Mean duration of intervention = 14.5 weeks. Mean estimated hours of total therapy = 29.5 weeks | TAU (n = 4), waitlist group (n = 10), non-specific control (n = 1). | PANSS (n = 12) SANS (n = 3) | PANSS-P, SAPS | SMD Subgroup analysis for type of intervention. | Small significant beneficial effect of MBE on NS in comparison with TAU & non-specific control intervention (SMD = -0.36, P = 0.00), high heterogeneity. Subgroup analysis found a beneficial effect of yoga on NS but high heterogeneity. Medium beneficial effect of Mindfulness on NS. |
| Vogel et al, 2019, Exercise | RCTs measuring the effect of PE on NS in SCZ, ≥ 18yrs, all settings Relaxation and exertion interventions also included. Peer reviewed studies in English. | 22 | DSM IV & V SCZ or related disorders | 1249 | − | Mind-body exercise (MBE), AE (light & moderate) or resistance training. | Mean duration of intervention = 12 weeks. Mean number of sessions = 33. | Active control (AC) = social skills training, psycho-education, relaxation, table soccer, occupational therapy, flexibility, toning and balancing exercises.  TAU | PANSS (n = 17) SANS (n = 5) | − | Hedges g. Subgroup analysis for separate effects of MBE & AE. I statistic for heterogeneity | Medium significant overall effect in favour of PE (g = 0.434, P < 0.001) high heterogeneity. MBE showed a statistically significant effect size in subgroup analysis (Hedges’ g = 0.461), with high heterogeneity. AE showed a small significant effect (g = 0.341), with moderate to high heterogeneity. PE only effective in comparison with TAU. |
| Polese et al, 2019, Psychological Therapy | RCTs with TAU control group, measurement of outcome with validated scales, Evaluation, pre- & post-treatment, Follow-up to 6 or 9 months. | 4 (studies included for NS only) | Treatment resistant psychotic disorder. | 800 (NS studies only) | − | Individual CBT | − | TAU | PANSS | − | SMD | No evidence for effects of CBT on NS (SMD = 0.075, P = 0.286). |
| Jia et al, 2020, Music | RCTs, music therapy as adjunct to APM TAU vs. APM or TAU alone, symptoms or QoL as outcome | 18 (11 of which were RCTs that looked at NS) | SCZ or related diagnoses as defined by any criteria | 1212 (764 in RCTs for NS analysis) | 34–68 | Music therapy (individual and group) | M = 33 sessions over a mean of 2.5 months | APM or TAU alone | BPRS, PANSS, SANS | Total symptoms, positive symptoms, depression, QoL | SMD, random effects model | Moderate effect of adjunct music therapy on negative symptoms (SMD = -0.61, P < .001) |
| Riehle et al, 2020, Psychological Therapy | RCTs or CTs psychological tx vs TAU or other psychological tx with NS as primary outcome, Minimum NS severity, & use of validated measures | 12 (6 of which included in RCTs only analyses) | DSM or ICD SCZ Spectrum Disorders | 516 (RCTs only analyses) | − | CBT | M = 31 weeks (range 8-72 weeks) | TAU (k = 4), CR (k = 2) | BNSS, NSA, PANSS, SANS, CAINS | Motivational/experiential NS, expressive NrS (BNSS, CAINS, SANS), functioning | Hedges’ g, random effects model | Small effect favouring CBT over TAU (g = ‑0.24, P = .05) for NS overall and motivational NS (g = ‑0.35, P < .05), but not for expressive NS (g = 0.10, P > .05). No difference between CBT and CR (g = 0.12, P > .05) for NS overall. |
| Hodann-Caudevilla et al, 2020, Psychological Therapy | RCTs, comparing Mindfulness Based Interventions (MBI) with wait-list or TAU | 10 (5 of which looked at NS) | DSM or ICD SCZ or related disorders | 1094 (506 in NS analysis) | 24–53 | MBI (Mindfulness Based Stress Reduction or MBCT) as adjunct to TAU | M = 13 sessions (range 8-32) | Active psychological tx or TAU | BPRS, PANSS, SANS | Overall symptomatology, Positive Smyptoms, Functioning, Mindfulness, Awareness of illness | SMD for pre-post change scores, random effects model | Small to medium effect of MBI on decreasing negative symptoms pre-to-post intervention (SMD = 0.40, P < .01) |
| Sabe et al, 2020, Exercise | DB RCTS: PE adjunctive therapy to APM vs a non-specific control condition or TAU. Minimum 3 weeks intervention & 8 h practice. English language. | 17 | 80% SCZ, 20% schizoaffective and other. Majority outpatients, all clinically able. | 954 | 18-65 M = 37.1 | Anaerobic exercise, aerobic exercise (AE), or non-specified exercise. | Non-aerobic interventions: mean = 17.6 wks, practice = 45 h, exercise = 60 min For AE interventions: mean length = 20.8 wks practice = 45.25 h, exercise = 47.9 min. | occupational activities, weight training. tabletop football or stretching (n = 6). TAU/ waitlist control group (n = 11) | PANSS (n = 15) SANS (n = 2) | SAPS | Standardised mean differences (SMD). Random effects model due to heterogeneity. | Small significant effect for PE on NS (SMD = −0.24, P = 0.01).  Significant difference for AE (SMD = −0.31, P = 0.006), but not non-aerobic exercise (SMD = −0.12; P = 0.50) |
| Burlingame et al, 2020, Psychological Therapy | RCTs. Aged ≥18. Groups were led by professional therapists. Studies employed a TAU, active or WLC group. Published 1990-2018. | 36 post treatment. 24 at follow-up | DSM, APA, or ICD. SCZ spectrum disorder | 4156 (total, not just negative) | M = 34 years | CBT, CR, IPT, metacognitive therapy; Multifamily groups; psychoeducation; social skills | 18 biweekly 75-min sessions composed of seven members. | AC, attention control, WLC and medication. | BPRS, PANSS | GAF scale, PANSS general psychopathology subscale, Rosenberg self-esteem scale, and hospitalisations. | Hedge’s g | Small beneficial effect at posttreatment (g = 0.27, P = 0.001) and follow up (g = 0.33, P = 0.004). |
| Jansen et al, 2020, Psychological Therapy | RCTs Intervention group included acceptance or mindfulness-based intervention. Aged ≥ 15 years, published or accepted in peer-reviewed journals. | 7 | DSM-IV or ICD-10 SCZ spectrum disorder or other psychotic disorder | 1268 | Total: 23.8-46.8 | ACT, MBCT, mindfulness-based psychoeducation, person-based CT, acceptance-based depression & psychosis therapy. | − | TAU - regular contact with case manager & psychiatrist, Waitlist or other AC. | PANSS, BPRS | CGI-SCH, hospitalisation, depression, QoL & social functioning, mindfulness skills, acceptance (psychological flexibility) | Hedges’ g | Significant small effects for NS (SMD 0.24, P = −0.03). There were significant differences between the two types of interventions, favouring Mindfulness. |
| Ma et al, 2020, Psychological Therapy | RCTs. Intervention with ≥ four sessions. Aged 18-65 years. Studies ≥30% comorbid neurocognitive or substance induced psychosis excluded | 2 (studies included for NS only) | ICD-10, DSM-5, or Chinese Classification of Mental Disorders Psychotic disorder | 158 (NS studies only) | M = 32.6 (studies included for NS only) | Any CBT-based family intervention involving family members ± service users | 6.75 weeks (studies included for NS only) | TAU, waitlist control or other psychosocial interventions. | PANSS, PSYRATS | SAI (Schedule for assessment of insight) | Mean differences | Significant effects for NS (MD = −4.35), but with high heterogeneity. Intervention had little effect on improving specific types of NS such as volition and self‐care ability. |
| Liu et al., 2021, Psychological Therapy | RCTs comparing a MBI to TAU, ≥ 18 yrs of age, outcome included total psychotic symptoms, positive or NS anxiety, or re-hospitalization | 9 (5 of which looked at NS) | SCZ spectrum disorders | 460 (in NS analysis) | 24–42 | Group MBI (Mindfulness Based Stress Reduction or MBCT) | M = 10 sessions over an average of 4 months (3 studies with 8 sessions over 2 months and 2 studies with 12 sessions over 6 months) | TAU | PANSS, SANS | Total psychotic symptoms, positive symptoms, anxiety, depressive symptoms, re-hospitalization | Hedges’ g, random effects model | Moderate effect favouring MBI over TAU in reducing negative symptoms immediately post treatment (g = −0.53, P < .001) and at short-term follow-up (g = −0.59, P < .001) |
| Lejeune et al., 2021, Psychological Therapy | RCTs: CR vs non-CR control, since 1980, English language, CR accounted for at least 50% of treatment time, | 73 (31 of which looked at NS) | ≥70% SCZ or schizo-affective disorder | 4594 (2581 in NS analysis) | M = 37 | CR ± interfacing with a PC, with drill-and-practice or drill-and-strategy model, training more than one cognitive domain, | M = 35 h over an average of 3 months and with 12 sessions/month | Passive and active control (no further description) | PANSS | MCCB domains, depression, positive symptoms, total symptoms, functional capacity, functional outcome, recovery | Hedges’ g, random effects model | Small effect favouring CR over non-CR treatments for negative symptoms (g = 0.16, P = .012, fail-safe N = 19) |
| Rodolico et al., 2022, Family interventions | RCTs comparing family interventions vs. TAU, at least 80% people with schizophrenia spectrum disorders | 90 (21 in NS analyses) | At least 80% people with schizophrenia spectrum disorders, studies including people with acute psychotic episodes were excluded | 10340 (2313 included in NS analyses) | M = 31.3 | ‘Family interventions’ | Median duration 52 weeks | Either other family intervention or TAU | PANSS, BRPS | relapse, overall symptoms, positive symptoms, expressed emotion, family burden, depressive symptoms, quality of life, adherence, functioning, tolerability, death | SMD, random effects model | Community-based care interventions (SMD = 0.32, P < .05), Family Pschoeducation with Family Behavioural Skills Training (SMD = −0.43, P < .05) superior to TAU, overall effect: SMD = −0.36, P < .01 |

NS = Negative Symptoms; ACT = Acceptance and Commitment therapy; CR = Cognitive Remediation; IPT = Integrated Psychological Therapy; INT = Integrated Neurocognitive therapy; MBCT = Mindfulness Based Cognitive Therapy; OT = Occupational Therapy; PE = Physical Exercise; SC = Supportive Care; SST = Social Skills Training; ST = Supportive Therapy; BNSS = Brief Negative Symptom Scale; BPRS = Brief Psychiatric Rating Scale; BSI = Brief Symptom Inventory; CGI-SCH = Clinical Global Impression Schizophrenia; Comprehensive Psychopathological Rating Scale (CPRS); NSA-16= Negative Symptom Assessment-16; PANSS = Positive and Negative Syndrome Scale; PSYRATS = Psychotic Symptom Rating Scale; SANS = Scale for the Assessment of Negative Symptoms; SAPS = Scale for the Assessment of Positive Symptoms; RE = Random Effects; FE = Fixed Effects; GAF = Global Assessment of Functioning; Wks = weeks; DB = Double Blind; SCZ = Schizophrenia; QoL = Quality of Life; APM = Antipsychotic medication

a. Range of mean ages is stated if this was only provided in the text
